# Testing silicone digit extensions as a way to suppress natural sensation to evaluate supplementary tactile feedback

**DOI:** 10.1371/journal.pone.0256753

**Published:** 2021-09-01

**Authors:** Leonard F. Engels, Leonardo Cappello, Anke Fischer, Christian Cipriani

**Affiliations:** 1 The Biorobotics Institute, Scuola Superiore Sant’Anna, Pisa, Italy; 2 Department of Excellence in Robotics & AI, Scuola Superiore Sant’Anna, Pisa, Italy; 3 Institute for Information Processing Technologies (ITIV), Karlsruhe Institute of Technology, Karlsruhe, Germany; Washington University in Saint Louis School of Medicine, UNITED STATES

## Abstract

Dexterous use of the hands depends critically on sensory feedback, so it is generally agreed that functional supplementary feedback would greatly improve the use of hand prostheses. Much research still focuses on improving non-invasive feedback that could potentially become available to all prosthesis users. However, few studies on supplementary tactile feedback for hand prostheses demonstrated a functional benefit. We suggest that confounding factors impede accurate assessment of feedback, e.g., testing non-amputee participants that inevitably focus intently on learning EMG control, the EMG’s susceptibility to noise and delays, and the limited dexterity of hand prostheses. In an attempt to assess the effect of feedback free from these constraints, we used silicone digit extensions to suppress natural tactile feedback from the fingertips and thus used the tactile feedback-deprived human hand as an approximation of an ideal feed-forward tool. Our non-amputee participants wore the extensions and performed a simple pick-and-lift task with known weight, followed by a more difficult pick-and-lift task with changing weight. They then repeated these tasks with one of three kinds of audio feedback. The tests were repeated over three days. We also conducted a similar experiment on a person with severe sensory neuropathy to test the feedback without the extensions. Furthermore, we used a questionnaire based on the NASA Task Load Index to gauge the subjective experience. Unexpectedly, we did not find any meaningful differences between the feedback groups, neither in the objective nor the subjective measurements. It is possible that the digit extensions did not fully suppress sensation, but since the participant with impaired sensation also did not improve with the supplementary feedback, we conclude that the feedback failed to provide relevant grasping information in our experiments. The study highlights the complex interaction between task, feedback variable, feedback delivery, and control, which seemingly rendered even rich, high-bandwidth acoustic feedback redundant, despite substantial sensory impairment.

## Introduction

Skilled use of our hands, whether to pick up a bottle of water or to perform brain surgery, depends crucially on somatosensory feedback from our fingers [1]. As experiments with anesthetized digits have shown, the most valuable information for successful grasping and lifting comes from discrete bursts of activity in the receptors of the fingertips [2–4]. When this feedback is missing, tasks as simple as grasping and lifting an object become slow and inefficient. Users of upper limb prostheses are missing haptic feedback from the prosthesis and rely on proprioception of the residual muscles. It stands to reason that providing supplementary feedback to prosthesis users would greatly increase dexterity and confidence in using their artificial hand [5, 6].

Yet, surprisingly few studies on artificial feedback for upper limb prosthesis users have indicated a functional benefit of the feedback [7]. It has been shown that simple feedback, such as a discrete stimulation upon contact, liftoff, replace, and release of the grasped object, can significantly improve prosthetic grasping [8, 9]. This form of feedback is called “DESC”, for “Discrete Event-driven Sensory feedback Control”. Yet, while these discrete feedback signals are certainly important for efficient grasping [2], they do not satisfy the yearning of prosthesis users to regain missing sensation [10]. Thus, it is paramount for researchers in the field to explore other types of supplementary haptic feedback if we are to eventually restore its full range.

Using invasive feedback, some studies have shown that continuous force feedback can lead to improved grasping control in functional tasks or every-day use [11–13]. However, this form of feedback is not available to the overwhelming majority of prosthesis users. Thus, much research today still focuses on non-invasive feedback [14–17].

Many of those studies have tried and failed to show a beneficial effect of non-invasive continuous force feedback (reviewed, for example, in [18]). Other studies showed beneficial effects only in strictly-controlled laboratory settings (e.g., virtual environments [19]), or only when vision and/or hearing were occluded [7, 20]. There are, however, some notable exceptions that could indeed show positive effects of non-invasive continuous force feedback [14, 21, 22] (and for the special case of amputees with targeted muscle-sensory reinnervation: [23, 24]). A recent study specifically addressing this problem concluded that “simple” feedback about the grasping force magnitude may not have beneficial effects to prosthesis users [20].

Continuous biofeedback on the control signal has been proven beneficial [25], either provided via electrocutaneous feedback [26] or auditory cues [15, 27]. However, biofeedback could be deemed somewhat unnatural, as the information is available without the prosthesis being in contact with an object and only indirectly related to hand-object interaction.

Interestingly, Johansson and colleagues found that during routine grasping, there is a very typical relation between grasp and load forces (e.g., [2, 28]). It is therefore an interesting but mostly untested question whether providing information about load in addition to grasp force would increase the benefit of continuous force feedback.

A recent study by Mastinu, Engels, and colleagues already provided a combination of grasp and lift force feedback with the intent of increasing motor coordination in prosthesis users. The results suggested that a combination of continuous force feedback and discrete event feedback may have positive effects on prosthesis handling [29]. However, due to the study design, only three amputee participants were tested, so it is unclear whether the result is generalizable.

Despite these advances, many studies on continuous feedback have unclear, ambiguous, or plain negative results, and it remains unclear why. We would like to suggest that there are some confounding factors, which mask potential benefit of the tested feedback. For example, many studies evaluate non-amputees, using a bypass to fit a robotic hand to the arm (e.g., [15, 30–33]). This extra weight could easily have an impact on movement of the arm and on muscle control of the robotic hand. Electromyographic (EMG) control itself could also have detrimental effects on performance because it is susceptible to environmental noise, and signal acquisition and processing cause delays in the control loop [34]. EMG control is especially difficult if study participants are new to this form of control, so that participants might be predominantly occupied with mastering control rather than paying attention to the supplementary feedback. On top of that, commercial hand prostheses are still very limited in dexterity and movement speed compared to unimpaired natural hands. More generally, there is still no consensus about which methods to use for providing feedback and assessing its effects and which metrics describe these effects most reliably. This also means that most studies differ in these aspects and are not directly comparable. All of these factors may impair the ability to design functional feedback, as well as its ideal use for improved prosthesis control.

We hypothesize that investigating supplementary feedback free from these constraints would paint a more faithful picture of the actual benefits and limits of different feedback methods. The ideal feed-forward tool free from the limits of prostheses would arguably be the entirely sensory deprived human hand, including all afferents mediating proprioceptive information. However, it may not be practical to anesthetize the hands of a large number of people for each new study on supplementary feedback. It has previously been shown that even slight hypoesthesia induced by wearing gloves already alters grasping behavior [35]. Based on this, we developed silicone digit extensions that affect grasping in similar ways to anesthesia by suppressing the response of tactile receptors in the fingertips [36]. Certainly, proprioceptive as well as mechanoreceptive afferents proximal to the fingertips, especially those with larger receptive fields (slowly- and rapidly/fast-adapting type 2 afferents), still mediate important grasping information, but the effect of decreased sensation in the fingertips is expected to be significant nonetheless [37–40].

In this study, we considerably impaired natural tactile feedback of limb-intact volunteers using silicone digit extensions similar to those we had already shown to have this effect previously [36]. Feed-forward control of the hand was essentially unaltered except for the necessary restriction of interphalangeal flexion of thumb and index finger.

In an attempt to increase the comparability of our results to those of previous studies, we assessed the effect of the impaired natural feedback as well as the supplementary feedback with a common pick-and-lift task used by many other studies and research groups (see, for example, [28, 29, 37, 41–43]). We hypothesized that we would be able to see increased grasp forces and extended grasp phase durations with impaired feedback [28, 37, 41, 42] and a positive effect of the supplementary feedback on these measures. We further stipulated that the maximum rate of force increases during loading would be lower without feedback, and the motor coordination between grasp and load forces would be negatively affected without natural or supplementary feedback. Since, however, the pick-and-lift task is rather simple and participants might have improved rather quickly through training, we added a second pick-and-lift task where the weight was varied. Natural feedback allows for a rapid adaptation to unexpected weight, but this mechanism is greatly impaired with diminished feedback [44], leading to higher uncertainty during grasping, which could increase the dependency on feedback cues [37]. We hypothesized that this would be improved again with supplementary feedback [29].

We provided three different kinds of non-invasive feedback–discrete, continuous, and a hybrid of the two–in an attempt to measure and compare the effect it would have on grasping with reduced finger-sensitivity. We hypothesized that supplementary feedback would allow participants to improve in routine and non-routine grasping, though the effect during routine grasping would likely be small [29]. The feedback was provided in the form of audio cues. Other non-invasive means of providing feedback, such as vibration or electrostimulation, have been tried in many previous studies (for a review, see for example [18]) but are limited in fidelity and bandwidth, and some introduce considerable delays. Visual cues could have provided similarly high bandwidth but would have interfered with visual attention.

By repeatedly testing the same participants over three consecutive days, we hoped to elucidate not only momentary performance differences between the study groups, but also the effect of habituation and learning. We also conducted a near identical experiment with the same feedback methods on a person with severe sensory neuropathy to evaluate the impact of our feedback strategies on a person who does not have any sensation in her hand and forearm and is thus used to grasping in “open-loop” (much like a prosthesis user).

Lastly, an additional possible confounding factor and important aspect of the participants’ experience is the task load. The task load is a subjective measure that factors in various aspects of a task’s demands, such as the cognitive and physical burden on each participant. Indeed, participants of an earlier study reported a considerable increase in perceived task difficulty with anesthetized digits [37]. Therefore, we used the well-established NASA Task-Load Index (TLX) questionnaire [45], and we expected to find a difference in task-load scores between feedback and no-feedback groups and hoped to be able to describe the contribution of the individual factors (cognitive, physical, etc.) to the overall workload (see also [14, 46]).

## Methods

### Participants

41 healthy adults with unimpaired hand and arm function and normal or corrected-to-normal vision (Age: 22–32 years, mean ± SD: 26 ± 2.4 years; 18 men; 4 left-handed) participated in the first experiment. One participant had to be excluded due to equipment failure, so data was processed from 40 participants. They were evenly and pseudo-randomly assigned to each of the four different feedback groups, resulting in 10 participants per group (S1 Table in S1 File). Group assignment was balanced for gender and music training, since neuroplasticity studies have shown that music training enhances sound processing capabilities not exclusively related to music, for example increased cerebral responses to subtle pitch changes and increased ability to extract meaning from sound and sound changes [47]. All participants were naïve to the purpose of the experiment.

Our volunteer for Study 2 was a 34-year-old woman with sensory impairment. Following a cartilage tumor at the level of vertebrae C1 and C2, GN lost nearly all perception in the right side of her body due to an incomplete posterior lesion of the spinal cord at level C1. GN has near normal sensitivity at the right shoulder and some diffuse perception that extends distally until the elbow, but she reports that this perception is very distinctly different from perception on her unimpaired left side. GN reported not to embody upper and lower limb on the right side, and she only managed to recover her ability to walk and to use her right hand after extensive rehabilitation. Her only way of estimating the applied grip force was through visual observation of the discoloration of her fingernail. Accordingly, we asked her to paint her fingernails with dark nail polish for the duration of the study.

To gain an understanding of her abilities, GN completed the Quick Disability of Arm, Shoulder, and Hand questionnaire (QuickDASH) [48] online [49], as well as the ABILHAND questionnaire for neuromuscular disorders [50, 51] (on the Rehab-Scales website of the Université catholique de Louvain, BE [52]).

All participants provided written informed consent prior to the start of the experiments, according to the Declaration of Helsinki. The study was approved by the ethics board of the Scuola Superiore Sant’Anna (approval number 2/2017).

### Setup

**Fig 1:**
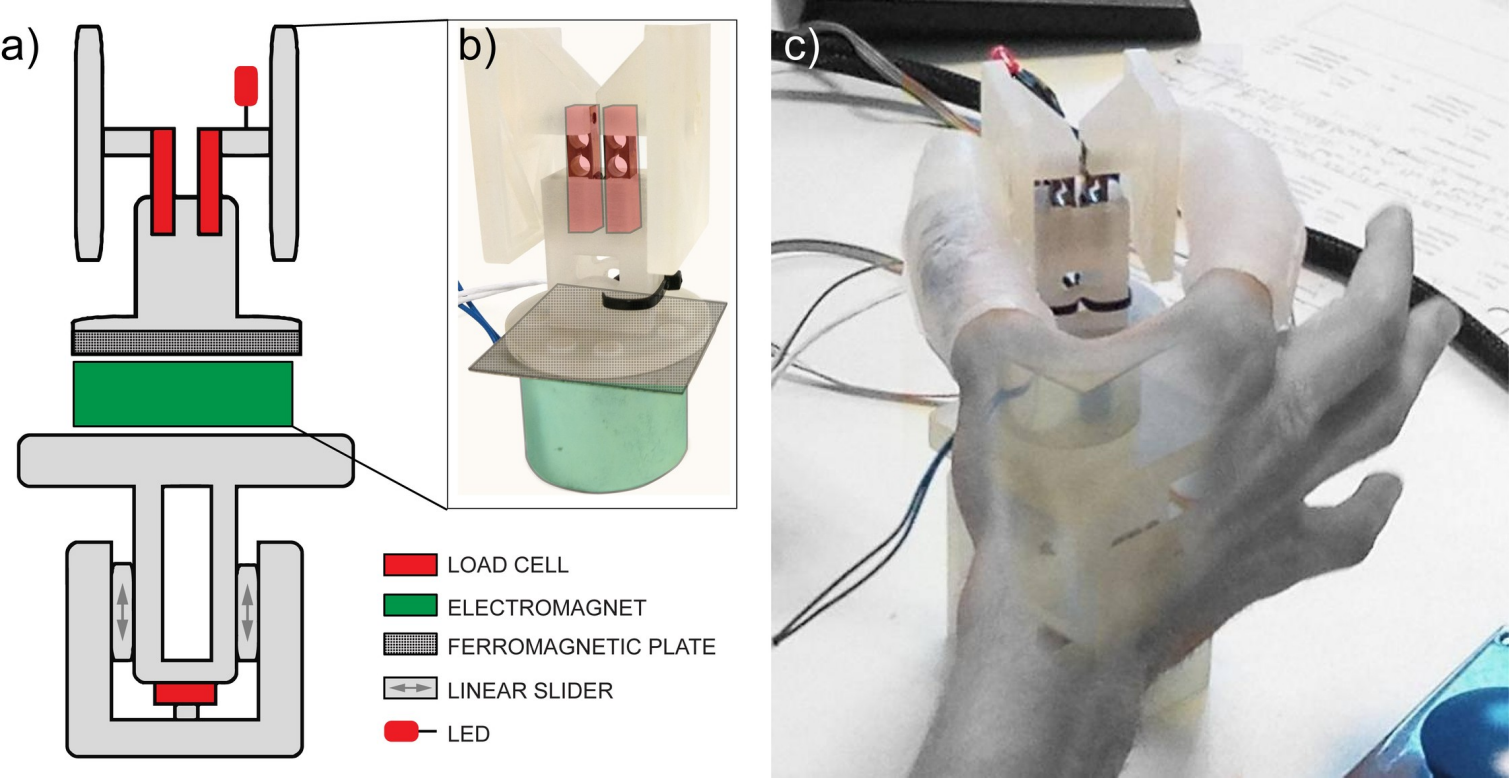
Sketch and photos of the instrumented test object with optional weight and the digit extensions. The weight was a non-ferromagnetic electromagnet that could be switched on without the participant’s knowledge and latched on to the underside of the object (green in sketch a). Two load cells measured the forces perpendicular to the grasping surfaces. The object stood on a platform that measured the lifting force (perpendicular to the table) until liftoff with another load cell. Sketch (a) also shows the LED. Photo (c) shows the object being grasped by a participant wearing the silicone digit extensions.

The participants sat comfortably in a height-adjustable chair in front of a desk with the experimental setup (S1 Fig in S1 File). On the desk, within reach of the participant, stood a small platform with the instrumented object on top. The platform measured the weight of the object and thus the change during loading (i.e., the load force) with a load cell (LSB200 model FSH00101, Futek, US). The object (weight: 80 g) consisted of two 3D-printed grasping panels affixed to two load cells (SMD2551-012, Strain Measurement Devices, UK) that measured the applied grasping force (Fig 1). On the bottom, a metal sheet enabled an increase of the object’s weight by allowing for a non-ferromagnetic electromagnet (ITS-MS-4027, Intertec Components GmbH, DE; weight: 190 g) to latch on to the object (increasing the object weight from 80 g to 270 g). Next to the platform, an indicator showed the participants the approximate height to which the object was to be lifted. A large push button was placed next to the object, and the participant pushed it at the start and end of every trial. A red LED affixed to the instrumented object turned on at the start of the trials and turned off after the object had been lifted off the platform for 2 seconds.

The object’s load cells were connected to a custom amplifier board, which was connected to a data acquisition board (PCIe 6259, National Instruments, US) together with the platform’s load cells, the LED, and the push button. All data were recorded by a desktop computer (Intel Core i7-6700 CPU, 3,40GHz, RAM 16GB) running Windows 10 Pro (Microsoft Corp., US) and Simulink (2019a, The Mathworks Inc., US) running in Simulink Desktop Real-Time with a sampling frequency of 1 kHz. The signals were processed in Simulink and relayed to Processing (v3.5.3, https://processing.org), a program that generated the appropriate audio feedback commands. These commands were sent to an Arduino Mega 2560 (Arduino, IT) via the data acquisition board, which translated the commands into audio output.

Audio feedback was delivered through standard commercial on-ear headphones (MDR-10RC, Sony, JP). All participants wore the headphones throughout the entire testing phase, regardless of whether they received supplementary feedback or not.

The custom digit caps employed in this study are an optimized version based on the preliminary design employed in our explorative work [36]. We empirically investigated the use of several silicone-based polymers to achieve the desired stiffness, in combination with different geometrical properties of the caps (i.e., thickness and length), which diffusely divided the contact pressure from the tips of the extensions onto many more mechanoreceptors than usual and decrease the activation of the receptors in the fingertips. Ergo, grasping forces are still relayed to the fingertips to a small extent, but those are not linearly related to the actual forces applied by the digit extensions due to the silicone’s compliance. In fact, we observed that with a shore hardness of 40 and a thickness smaller than 3 mm, the digit caps would not produce the desired effect, while a thickness larger than 5 mm would make the finger motion too clumsy. A smaller shore hardness would have required larger thickness, while a larger hardness would have made the caps very uncomfortable to wear for the duration of the study. On the other hand, their length was optimized to separate the contact point from the fingertips without the risk of bending, which may have reduced the dexterity of the finger. The caps were available in two different sizes (thickness 3–5 mm), covered the entire thumb and index finger, and extended ca. 25 mm beyond the fingertips (total length of the caps: 90 mm for the index and 70 mm for the thumb). The digit extensions provided no considerable extra weight (weight: 25–35 g). Proprioception and feed-forward control of the hand were not altered except for the restraint of thumb and index flexion at the interphalangeal joints.

### Experimental procedure

The participants were instructed about the experiment in written form. The experiment was divided in two tasks: Task 1 was a simple routine lifting task, and Task 2 tested the participants’ adaptation to unforeseen weight changes of the object from one trial to the next. During both tasks, participants were instructed to approach, grasp, and lift the object ca. 5 cm off the table in one fluent motion and at a comfortable pace. Each grasp-lift-replace trial was preceded and followed by the press of a large push-button on the table next to the object. The grasp was always a precision grasp between thumb and index finger, and the silicone digit extensions were placed over both fingers of the dominant hand during all trials.

In Task 1, participants first lifted the low weight 20 times and then the higher weight. Participants were made aware of the change in weight.

In Task 2, participants were instructed that the weight could vary between lifts, but it would stay the same weight for a short while after every change. The Simulink program changed the weight randomly every 4 to 6 lifts by turning the electromagnet on or off, for a total of 12 weight changes (theoretical minimum number of trials: 52, maximum: 78).

Each experimental task was followed by the NASA Task Load Index (TLX) and a custom extension of that questionnaire regarding the feedback, in the same format as the TLX (see Supplementary methods in S1 File).

Participants were allowed to take breaks at any point if they so wished but were not allowed to remove the digit extensions until the end of each task.

#### Study 1–Participants without limb impairment

**Fig 2:**
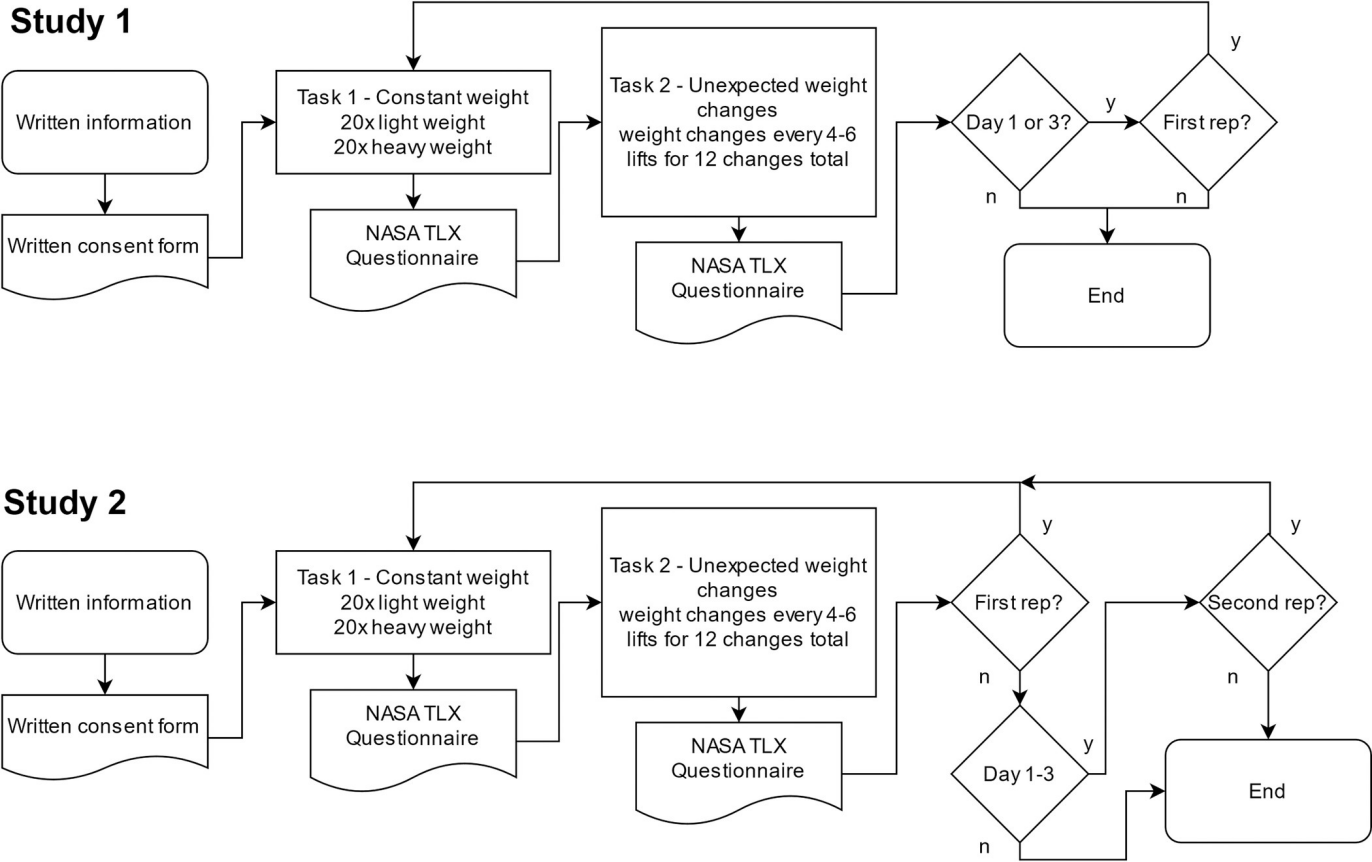
Flowchart of the experimental procedures of Study 1 and 2. In Study 1, participants performed both tasks twice (without and with feedback according to group) on days 1 and 3 but only once (with feedback) on day 2. In Study 2, GN performed the same tasks three times (without, with, and again without feedback) on days 1–3 and only twice (without feedback) on day 4 (control condition). TLX = Task Load Index (including our custom extension of the questionnaire).

All participants took part in the experiment on three consecutive days; in total, the experiment lasted ca. 2 hours and 15 minutes. On day 1 and 3, participants did each task twice, once without artificial feedback, and once with feedback according to their experimental group (i.e., the “NOFB” group did not receive supplementary feedback in this second condition either). On day 2, participants only performed each task once, with feedback according to their experimental group (Fig 2).

#### Study 2–Participant with sensory neuropathy

For participant GN, the tasks were identical, but she performed the tasks without the digit extensions and tested each feedback only for one day. On days 1 to 3, she repeated Tasks 1 and 2 once each without any supplementary feedback, once each with feedback, and then again without feedback—a typical ABA scheme for single case studies. On day 4, GN performed Tasks 1 and 2 twice without any supplementary feedback (Fig 2).

### Feedback

The four different groups received different feedback during the “feedback on” phases of the experiment. One of the groups acted as control and simply repeated the tasks the same number of times as participants in the other groups, but without ever receiving additional feedback (“NOFB”).

For the other three groups, supplementary sensory feedback was provided as mono audio cues. The amplitude of the audio feedback was set to a comfortable level for each participant on day 1 and kept constant throughout the study. The duration and frequency varied according to the feedback group. The feedback was turned on and off discretely, and the frequencies were pure sine waves with discrete steps from one to the next.

The discrete or “DESC” feedback group received auditory cues upon contact with the object, liftoff, replace, and release of the object for 0.07 s at a frequency of 1174 Hz (D_6_ in idealized standard piano tuning).

As described above, we hypothesized that providing information about lifting as well as grasping forces would be beneficial. Seeking to exploit the typical relation between grasp and lift forces in routine grasping [28], the next group was consequently provided with continuous feedback (“CONT”) related to the ratio of grasping and lifting force; that is, upon contact, grasping force was divided by the lifting force. The resulting force ratio was mapped to a frequency range of 220 to 1174 Hz (A_3_ to D_6_) in half-tone steps (= 30 steps). The lowest frequency (220 Hz) corresponds to the minimum necessary GF to lift the object. The highest frequency (1175 Hz) corresponds, for example, to a GF of >11 N before starting to lift the object (i.e., a GF far exceeding the necessary).

The last group received a hybrid feedback (“HYBR”), meaning discrete auditory cues at contact, liftoff, replace, and release, like the discrete group, in addition to continuous force feedback while the object was grasped, like the continuous group.

The logic behind each feedback was not explained to the participants to prevent any biasing. We hypothesized that the participants would intuitively use the feedback after some exposure. The extended TLX measured the conscious understanding of the feedback.

### Data analysis

#### Extracted metrics

Data were processed in Matlab (2017b, The Mathworks Inc., US). From each trial, we extracted a number of metrics, including but not limited to the standard metrics used in previous analyses of grasping [2, 28, 29, 36, 42, 43, 53–55]. For conciseness, only the following three metrics are presented here in detail:

- duration of the load phase: the duration in seconds from the moment the participant starts applying a load force (upwards) until the moment the object lifts off the platform; it extends significantly when natural feedback is suppressed [28, 37, 41]
- peak grasp force (GF) rate during the load phase: the maximum rate of GF applied onto the sides of the object during the load phase (the rate was obtained by calculating the time derivative of the force and subsequently applying a moving average filter with a 20 ms window and 10 ms overlap)
- grasp force-load force (GF-LF) delay: the time difference between load force (LF) reaching 50% of the maximum LF and GF reaching the same force [8, 9, 29].

#### Data processing

In Task 1, the first five trials of each set of 20 were discarded as “training” data to ensure we would only analyze true “routine grasping”. Outliers, defined as values that were more than three scaled median absolute deviations away from the median, were removed from each remaining set of 15 trials. We then took the median of each set to obtain a singular value per metric, representing the performance of each subject in each experimental condition. These values were then further analyzed in SPSS (version 20, IBM Corp.) or Matlab 2017b.

In Task 2, all trials of each set were scanned for outliers. We then took the median of each “weight change condition” per set, i.e., all the trials preceding a weight change in a particular direction, the weight change trial, and the one following that. These were then further analyzed in SPSS or Matlab.

#### Statistical analysis

Datasets were tested for normality of the distributions with the Shapiro-Wilk test. Normally-distributed datasets were tested for homogeneity of variances with Levene’s test, and, depending on the comparison, for sphericity with Mauchly’s test and for equality of covariance matrices with Box’s M test. If data fulfilled the requirements, a mixed ANOVA was performed to assess the effects of feedback condition and time, as well as their interaction. If the assumption of sphericity was not met, a correction was applied according to the value of epsilon. If the other requirements were not fulfilled, we evaluated if a log_10_ transformation would allow the data to be analyzed with a mixed ANOVA; if not, we proceeded as follows.

#### Task 1

If distributions were normal, but variances were not homogeneous, a one-way repeated-measures ANOVA (3 days) or paired t-test (2 days) was performed to assess the effect of time; and for each day, Welch’s ANOVA with a Games-Howell post-hoc test was performed to assess the effect of feedback. When a repeated-measures ANOVA was performed, distributions were tested for sphericity as described above.

If the data was not normally distributed, we tested for differences between feedback conditions with a Kruskal-Wallis test for each day and Bonferroni-corrected Mann-Whitney U tests for post-hoc comparison. To test for differences in time, we used Friedman’s test (to compare 3 days; ensuring that the distributions were mostly comparable), or a Wilcoxon test (to compare 2 days; ensuring that the distributions of the differences were approximately symmetrical). Significant Friedman tests were followed up with Bonferroni-corrected Wilcoxon tests.

#### Task 2

In task 2, the “preceding”, “weight-change”, and “following” trials were individually compared between groups. If the data were normal and variances were homogeneous, a one-way ANOVA was used, followed by Tukey’s honestly-significant difference post-hoc test in case of significance; if variances were not homogeneous, Welch’s ANOVA was used, potentially followed by a Games-Howell post-hoc test. Non-normal data were compared with a Kruskal-Wallis test, followed by Bonferroni-corrected Mann-Whitney U tests in case of significance.

Furthermore, the three trials were compared within each feedback group using a repeated-measures ANOVA for normally-distributed data (corrected if the assumption of sphericity was not met, followed by paired t-tests in case of significance). If data were not normal, a Friedman test was used, followed by Bonferroni-corrected Wilcoxon tests.

All pairwise comparisons were Bonferroni-corrected (p-value adjustments that would result in values larger than 1 are denoted as “p = 1.0”).

All values are reported as median (interquartile range) unless otherwise noted.

In study 2, we only collected data from a single participant, so no statistical analysis was performed. Instead, we plotted time series of all data for visual inspection. To further aid inspection of the data, we also calculated the “Percentage of Non-overlapping Datapoints” (PND) between two adjacent phases, and the “Stability” of the data, meaning the percentage of datapoints that are within 15% of the median.

## Results

There were no meaningful differences between any of the feedback groups. We therefore deem it meaningless to report all the metrics. In the following, we will shortly describe the results of the few chosen metrics to illustrate this outcome. The results of the questionnaires are reported in the supplementary materials. However, there was no considerable difference in the overall workload between feedback and no feedback for any of the groups on any of the days, neither for Task 1 nor Task 2 (S7 Fig in S1 File). There was also no obvious difference between the two tasks.

### Grasping tasks

#### Study 1

*Task 1*. Whenever it was possible to use a mixed ANOVA, the result showed that there was no significant interaction between feedback groups and time. Accordingly, only the main effects of group and time are reported in the following. All mixed ANOVAs were done on log-transformed data, as no data conformed to all the requirements without transformation.

#### Duration of the load phase

**Fig 3:**
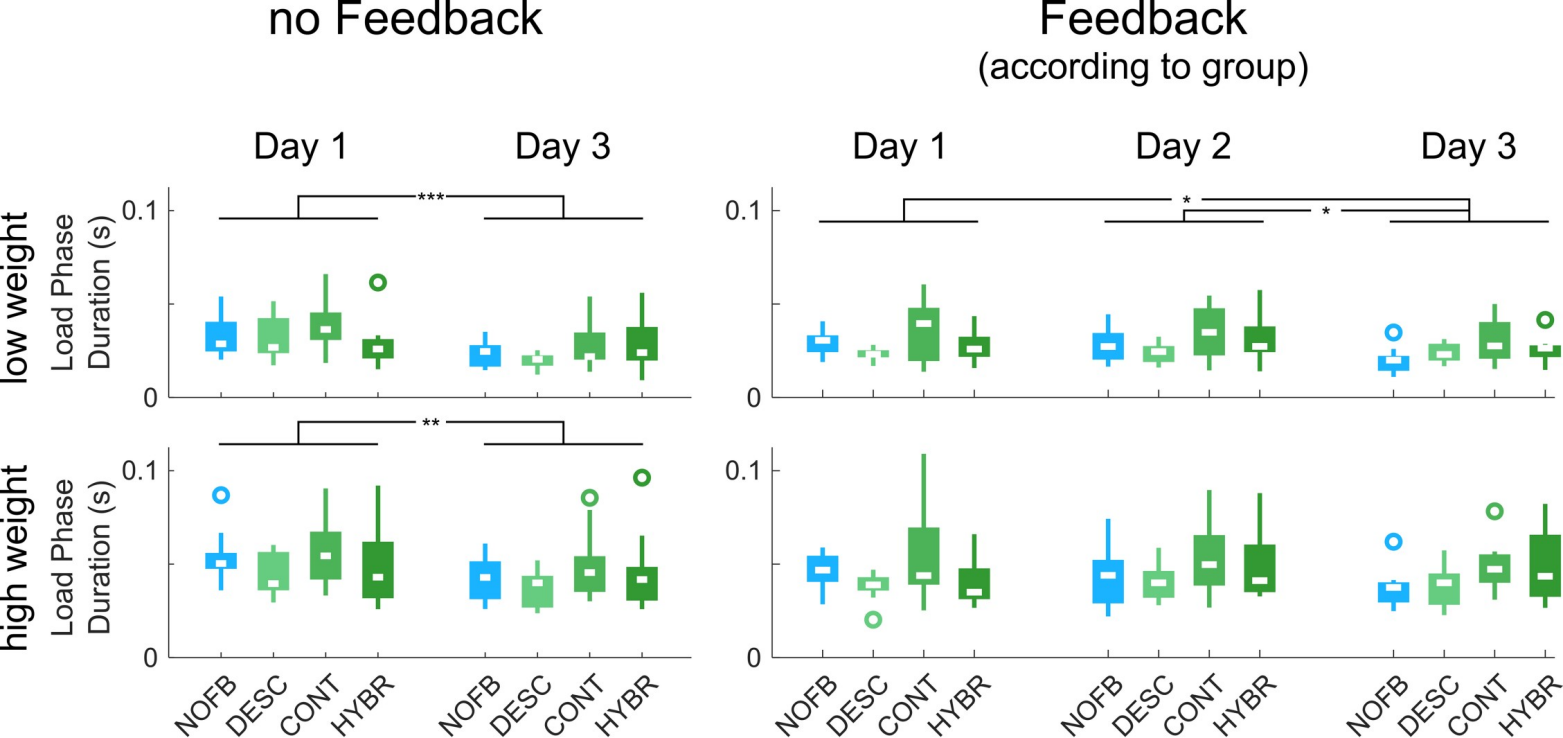
Duration of the load phase in Task 1 of Study 1. The graphs on the left display performance without any feedback, the graphs on the right with feedback according to group (the “NOFB” group, in blue, never received supplementary feedback). As can be seen, there are significant differences in the performance between days (irrespective of feedback group) but not between groups. Boxplots display medians, 25^th^ and 75^th^ percentiles; the whiskers denote the most extreme datapoints that are not outliers; circles denote outliers. * = p<0.05, ** = p<0.01, *** = p<0.001.

Difference between days (Fig 3): There was a significant difference in the duration of the load phase between days one and three when all participants received no supplementary feedback (low weight: F(1, 36) = 26.10, p < 0.001; high weight: F(1, 36) = 9.106, p = 0.005). When feedback was provided according to the experimental groups, there was a difference only with the low weight (repeated-measures ANOVA: F(2, 78) = 6.824, p = 0.002) from day 1 to day 3 (p = 0.035) and day 2 to day 3 (p = 0.01) but not between days 1 and 2 (p = 1.0). With the high weight, however, there was no significant difference between days (Friedman test: Χ^2^(2) = 0.340, p = 0.844). For representative example time series of the load phase duration for Tasks 1 and 2 see S2 and S3 Figs in S1 File.

Differences between groups (Fig 3): Regardless of whether feedback was provided or not and whether the weight was high or low, there were no significant differences between groups (all Bonferroni-corrected p-values > 0.05).

#### Peak rate of grasp force during the load phase

Difference between days: The peak grasp force rate during the load phase did not differ between days when no feedback was provided (low weight: F(1, 36) = 3.598, p = 0.066; high weight: F(1, 36) = 0.235, p = 0.631). With feedback according to group, there was a significant difference between days 1 and 2 and days 1 and 3 with the low weight (Friedman test Χ^2^(2) = 12.200, p = 0.002; post-hoc comparison: day 1 vs. day 2, p = 0.009; day 1 vs. day 3, p = 0.003; day 2 vs day 3, p = 1.0). With feedback and the high weight, there was no difference between days (Friedman test Χ^2^(2) = 0.200, p = 0.905).

Difference between groups: Without feedback, there was no difference between groups for either weight (low weight: F(3, 36) = 1.455, p = 0.243; high weight: F(3, 36) = 1.119, p = 0.354). With feedback as well, there were no significant differences between groups, regardless of object weight (Kruskal-Wallis tests for each day, all p > 0.05).

#### Delay between grasp and load force

Difference between days: Without feedback, there was a significant difference between days 1 and 3 (low weight: F(1, 36) = 65.263, p < 0.001; high weight: Wilcoxon z = -4.395, p < 0.001). With feedback and the low weight, there was again a significant difference between days (F(2, 72) = 5.058, p = 0.009, day 1 vs. day 3: p = 0.021). However, with feedback and the high weight, there was no significant difference between days (F(2, 72) = 2.013, p = 0.141).

Difference between groups: Without feedback and with the low weight, the difference between groups was significant (F(3, 36) = 4.463, p = 0.009). Tukey’s HSD post-hoc test revealed a significant difference between NOFB and CONT (p = 0.033), and DESC and CONT (p = 0.010), but not between the other groups (all other p > 0.05). Without feedback and the higher weight, there also seemed to be a significant difference between groups, but only on the first day (H(3) = 8.058, p = 0.045). Pairwise comparison, however, revealed no significant differences between groups (all p > 0.05).

With feedback and the low weight, there was no significant difference between groups either (post-hoc pairwise comparisons all p > 0.05). With the high weight, there was a difference between DESC and CONT (F(3, 36) = 3.47, p = 0.026; DESC vs. CONT: p = 0.047). All other differences between groups were non-significant.

*Task 2*. As with Task 1, there are essentially no differences between groups in any of the considered metrics. There are, however, some exceptions regarding the DESC and CONT group: on day 1, with feedback, there is a significant difference between the DESC and CONT group in the weight-change trials from low to high weight (p = 0.018). And in the GF-LF delay metric, we find a difference between these groups on day 2, with feedback, in the weight change trials from high to low (p = 0.031), and on day 3 with feedback in the trials following a weight change from low to high weight (p = 0.004).

In all of the metrics, on all days, with or without feedback, and with both weights, there is a significant difference between the weight change trial and the one preceding it (all p < 0.05), suggesting that the grasp was adapted to the changing weight (the only exception being the peak rate of grasp force during the load phase with feedback when switching from a high to a low weight on day 2 (p = 0.066); but the difference is highly significant on days 1 and 3).

In many cases, the trial following a weight change is significantly different from the trial preceding the weight-change.

The weight-change trial and the one following are never significantly different from one another (all p > 0.05, most p = 1.0).

#### Study 2

We repeated the same tasks in a slightly different experiment with a single volunteer, GN, with extensive sensory neuropathy in the right upper limb. As in study 1, there seem to be no considerable differences in performance with and without feedback.

To understand GN’s manual ability, we also asked her to fill out the QuickDASH and ABILHAND-NMD questionnaires. In the former, she reached a score of 13.6, and in the latter, the score was 34 and the patient measure 4.17 ± 0.75 (SE) logits. This suggests that GN is very capable of using both hands dexterously, despite her neuropathy.

*Task 1*. With the high weight but not the low weight, the load phase duration was slightly longer with the DESC feedback than without (S4 Fig in S1 File). Related to that, we saw that the peak GF rate during the load phase was also lower with DESC than without.

There was no apparent difference in performance with the CONT feedback, compared to without.

With the low but not the high weight, the GF-LF delay seemed considerably lower with Hybrid feedback than without feedback (S5 Fig in S1 File). There was no apparent difference with HYBR in any of the other metrics.

Performance without feedback did not change much over the four days of testing (S4 Fig in S1 File). This confirms that the task was true routine grasping, and that the subject had no difficulties performing it.

**Fig 4:**
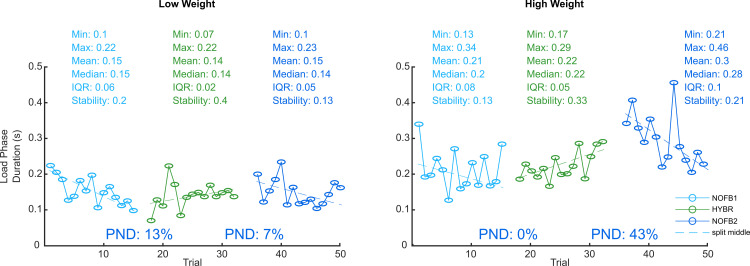
Time series of the load phase duration with and without HYBR feedback in Task 1 of Study 2. The trials without feedback are presented in blue, the trials in green are with HYBR feedback. This example shows that there is an almost complete overlap of trials without and with feedback. PND = Percentage of non-overlapping datapoints between two adjacent phases (in the desired direction, i.e., “HYBR lower than NOFB”); IQR = interquartile range; Stability = percentage of datapoints that are within 15% of the median.

Fig 4 displays the duration of the load phase on a trial-by-trial basis an example of the development of GN’s performance with and without HYBR feedback. We found that there was no tangible difference with any of the feedback methods. Indeed, when looking at the time series, we found strong overlap between the trials with and without feedback. Most PNDs were well below 20%.

To see whether the digit extensions led to similar grasping behavior as complete sensory neuropathy, we also compared the median values for the three metrics during routine grasping. The median (IQR) load phase with the low weight lasted 0.103 (0.057) s for the digit extension group and 0.151 (0.064) s for GN. With the high weight, it lasted 0.172 (0.075) s for the former and 0.237 (0.077) s for GN.

The GF-LF delay with the low weight was 0.143 (0.099) s for the digit extension group and 0.144 (0.066) s for GN. With the high weight it was 0.155 (0.112) s for participants of study 1 and 0.143 (0.035) s for GN.

And the peak GF rate during loading was 34.50 (21.93) N/s for the former and 22.37 (9.10) N/s for GN with the low weight, while with the high weight, it was 63.65 (39.47) N/s for the former and 46.0 (8.73) N/s for the latter.

The digit extensions seem not to affect these grasping metrics as much as complete neuropathy does, but since the interquartile ranges are all overlapping, the effect appears comparable.

*Task 2*. In the second task, there were no apparent differences between grasping without feedback and grasping with any of the feedback methods in any of the considered metrics.

When looking at the time series, the overlap between trials with and without feedback was even stronger than in the first task. Most PNDs were below 5% (e.g., S6 Fig in S1 File).

## Discussion

In this study, we used silicone digit extensions to suppress natural sensory feedback from the fingertips of otherwise unimpaired volunteers. We compared the performance in a pick-and-lift task with this reduced feedback to performance with additional auditory feedback about the interaction of digit extensions and the test object. We then repeated a near identical experiment with the same three kinds of auditory feedback with a volunteer with extensive sensory neuropathy.

We had hypothesized that providing supplementary sensory feedback would enable the participants to improve their performance in routine and non-routine grasping. The metrics we assessed have been used previously to describe grasping (e.g., [29, 44]). More detailed analyses are possible and have been done in the past, including many other metrics (see Methods - Extracted metrics), but since our analysis did not reveal any meaningful differences between the groups, we limited ourselves to reporting three metrics for sake of brevity.

We assumed that at least discrete feedback would prove beneficial. In fact, the discrete feedback that we provided is based on the well-known DESC hypothesis [2] and has been shown to allow limb-normal volunteers and amputees to significantly improve their grasping abilities in similar tasks when using a prosthesis [8, 9]. In these studies, discrete feedback was delivered through a vibrotactor on the forearm. However, we could not report any effect of the feedback on grasping performance. This would suggest that the way feedback was provided in our study (through audio cues) impeded its use or, perhaps more likely, that the results from the studies on the DESC hypothesis are not transferable to our study because of the substantially different end effector (prosthesis versus hand).

However, previous studies have also shown that providing continuous audio feedback can influence grasping behavior beneficially in virtual tasks [56] and when controlling a robotic hand through a Data Glove [46], or myoelectrically [15, 27], for example. This would imply that it is not the auditory system in general that is not suitable to interpret information relevant for grasping. Instead, it could be the specific encoding we used, mapping the continuous forces onto a large range of discrete half-tone frequency steps. In [15, 27] and [56], the amplitude of the audio cues rather than the frequency was changed continuously, which might have been somewhat more intuitive. Gonzalez and colleagues [46] provided audio feedback in the form of only five major triads, with three full-tone steps and one half-tone step. Here, perhaps the vastly smaller amount of information was easier to understand and use than the 30 half-tones steps in our study. Importantly, though, all of these studies differed not only in the implementation of audio feedback but also in the type of feedback: rather than providing information about force, these studies provided information about position, which was available even before contact with the object (see also the promising biofeedback approaches in [25, 26] but compare to [15]).

Audio feedback as substitute for tactile sensation is arguable rather unintuitive, but due to its large bandwidth—only comparable to visual feedback—it has found many previous applications in research on sensory feedback [46, 56–58]. While we do not actually suggest hearing as a suitable modality for general prosthesis feedback, in the specific case of bone-anchored prostheses, audio feedback could easily be provided through vibrations on the bone-implant and perceived as sound [59].

It could, of course, also be argued that the digit extensions do not, in fact, reduce tactile feedback from the fingertips enough to make participants rely on the artificial audio feedback. Although we could show that similar silicone digit extensions significantly alter grasping in a previous study [36], this change may not be primarily due to a considerable reduction in sensation from the fingertips but rather due to significantly altered movement mechanics of the finger (e.g., it could not be bent and was unnaturally extended). It seems likely that the silicone digit extensions in the present study necessitated only minor adjustments to the internal model of normal grasping [60, 61]; perhaps this change was more pronounced in the previous digit extension study, as the extension design was slightly different.

While it has been shown previously that covering the fingertips to reduce sensation has a significant impact on grasping [35], and more proximal mechanoreceptors and proprioceptors of the hand are much less sensitive and their feedback much less precise [3, 40, 41], it could be argued that this information combined was enough to perform both tasks in the present study. To be able to tease out the actual contribution of proprioception, future experiments could involve measurements of movement trajectory and/or recordings of proprioceptive afferents. Stretch-sensitive skin mechanoreceptors in the hand could have provided detailed information about finger position [62] but this should not have had an effect of force scaling as the object was not deformable. The responses of muscle spindle afferents, on the other hand, are complex during active movement [63] and a detailed discussion of their contribution would greatly surpass the scope of this manuscript. However, here we also showed that routine grasping performance with the digit extensions and without feedback was only slightly better than that of GN without feedback, who has no remaining sensation in her hand and forearm, including proprioception. This would argue against the hypothesis that the digit extensions did not suppress natural feedback sufficiently to make supplementary feedback meaningful. Indeed, if the performance in study 1 were based primarily on proprioception, we should see greater differences between studies 1 and 2.

In line with previous experience (e.g., [14, 16, 29]) and with the recommendations by Sensinger and Dosen [7], we assessed the feedback over several sessions. As expected, performance increased over days, even for the simple feed-forward task of routine grasping; this exemplifies the importance of testing over several days to measure the effect of learning. However, as expected, Task 1 may not have been challenging enough to necessitate the use of supplementary feedback, as evinced by the lack of significant differences between feedback and no-feedback groups [29]. The only differences we did find were likely not due to the fact that one feedback was more informative than another, but rather that, despite the random group assignment, the performance of the participants in the CONT group seemed worse overall, regardless of whether feedback was provided or not.

For that reason, to provoke the use of sensory feedback mechanisms, Task 2 was designed to be more challenging, forcing participants to quickly adapt to changing weights [29, 44, 53, 64]. The analysis showed that weight changes indeed provoked significantly different grasping behavior most of the time, meaning that the difference in weight was substantial enough. However, the participants adapted to the new weight within one trial, even when no supplementary feedback was provided. Previous studies suggest that the adaptation would not happen so quickly with completely anesthetized digits, supporting the notion that the digit extensions did not block natural feedback sufficiently [37, 41, 42]. Yet also in the second study, where the participant, GN, had no remaining sensation in the hand, there seemed to be no clear difference between Task 2 with and without feedback. According to Sensinger and Dosen [7], it seems reasonable to assume that GN used her extremely well-developed feed-forward model for solving the grasping tasks–just like an experienced prosthesis user would. Indeed, this may point yet again to the importance of extended training with a novel feedback system. GN, just like many prosthesis users in other studies, had very limited time to explore the feedback and develop a new internal model based on the additional information provided by it. It is also possible that the subjects in study 1 were exposed to the two weights so repeatedly in Task 1 that they were able to grasp and lift them successfully in Task 2 after only a single trial, meaning they would have developed a sufficient feed-forward model of the task, similar to GN. However, it also seems reasonable to assume that at least the participants of study 1 that are used to relying on feedback-based grasping when the grasped object behaves unpredictably, should have incorporated any useful information from the feedback readily in Task 2 [37]. Indeed, we would imagine that persons who are used to relying on feedback can integrate new feedback more easily than those who have trained for many years to be independent of it. Future studies should allow neuropathy or amputee participants to engage with the feedback for many subsequent days, rather than hours–something “home-use” studies have been very successful with (e.g., [8, 12, 23, 65]). That this was not the case may suggest that the task was still too easy. Perhaps the >3 fold increase in weight from light to heavy was still too small [3, 44], although we described above how the behavioral change in response to the changing weight was indeed significant, and the weight ratio is comparable to [44] despite the actual weights being smaller. The minimum weight in our study was limited by the necessary components in the object (load cells, big enough grasping surfaces) and the maximum weight was constrained by the compliance of the silicone digit extensions. Future studies might use heavier objects similar to [44]. A larger total weight difference may have also led to a larger difference in perceived workload of the two tasks. This leaves the possibility that our particular implementation of the feedback was not informative enough to be useful.

It is clear that one of the main tasks of the central nervous system is to integrate information coming from different sensory modalities, thus minimizing the uncertainty of sensory signals [66, 67]. It is also evident that information from supplementary feedback would only be useful if the uncertainty of its informational content were lower than that of the remaining natural feedback [68, 69]. Thus, Sensinger and Dosen [7] also recommend assessing the feedback strategies using psychometric tests. That way we could have estimated the uncertainty with which each participant perceived and interpreted the feedback (and thus gained indications for how much weight the participants would attribute to each feedback strategy [69]). We checked that the stimulus was clearly perceivable, and we believe that all participants could identify semitone frequency steps, but the perceived loudness of the stimulus and the ease with which the frequency steps could be discerned may indeed have varied between participants and between sessions [70]. For maximum efficacy of the feedback, the stimulus would have needed to be adjusted to each individual, as described recently by Karakuş and Güçlü [70] for vibrotactile stimuli. The procedure described therein consists of absolute detection-threshold measurement, identification of psychometric function, subjective magnitude assessment, and the determination of equal magnitude levels for different frequencies. They also describe how this arguably long procedure only needs be completed once per subject and can then be adapted to changing conditions by a much shorter recalibration procedure. In addition, the experimental conditions without feedback could have benefited from psychophysically-adapted masking noise. While such calibration could have ensured that each participant perceives the feedback clearly and at the same threshold throughout the entire range, we do not believe this would have significantly altered the presented results. Regardless of the exact reason, we can conclude that the supplementary feedback in this study failed to provide more information than the remaining natural feedback.

We do believe that the attempt to suppress natural sensory feedback with silicone digit caps was straight forward and merited exploration. Consequently, the relevance of this study lies not just in its results, which add to the vast evidence that supplementary feedback remains an elusive phenomenon. Instead, we hope that other researchers can relate to our reasoning for devising these experiments and improve on the aspects in our study–task design, feedback logic, feedback delivery–that seem to have created more questions than they have answered.

## Supporting information

S1 File Supplementary materials—contains supplementary methods and results about the questionnaire. (DOCX)

S2 File (ZIP)
