# Supplementary material for: Testing silicone digit extensions as a way to suppress natural sensation to evaluate supplementary tactile feedback
Source: PLoS One. 2021 Sep 1;16(9):e0256753. doi: 10.1371/journal.pone.0256753 (PMC8410127; doi:10.1371/journal.pone.0256753)
Supplement: S1 File — (DOCX) [file pone.0256753.s001.docx]

# Supplementary materials for „Testing silicone digit extensions as a way to suppress natural sensation to evaluate supplementary tactile feedback“

# by Leonard F. Engels, Leonardo Cappello, Anke Fischer, Christian Cipriani

## Supplementary methods

**S1 Table.** **Group assignment of participants of Study 1.**

| **Feedback Group** | **NOFB** |  | **CONT** |  | **DESC** |  | **HYBR** |  |
| --- | --- | --- | --- | --- | --- | --- | --- | --- |
| **Musical education** | **No** | **Yes** | **No** | **Yes** | **No** | **Yes** | **No** | **Yes** |
|  | F | F | F | F | M | M | M | M* |
|  | M | F | F | F | F^#^ | M | F | F |
|  | M | F | M | M | F | F | F | F |
|  |  | M |  | M |  | F | F | M |
|  |  | M |  | M |  | F | M | F |
|  |  | M* |  | M |  | F |  |  |
|  |  | F |  | F* |  | F |  |  |
|  |  |  |  |  |  | M* |  |  |

This table shows the gender of each participant in each group (F = female, M = male), as well as whether they have received musical education or learned to play an instrument, which could potentially affect how the audio feedback is perceived.

* = left-handed

# = excluded

### Questionnaire

To attempt to protocol the subjective experience of the participants during the experiment, we employed the NASA Task Load Index (TLX) to measure the task load of each task, and we expanded it by four questions that aimed to specify the participants’ experience with the feedback. The TLX is a well-established questionnaire developed by NASA researchers in 1988, which aims to compute an overall workload score for any given task by asking participants to rate the load on six specific dimensions: Temporal, Physical, and Mental demand, Frustration, Effort, and Performance [1,2]. Each is rated on a scale from 0 to 100.

We extended the questionnaire by four items pertaining to the received feedback: Comprehension, Benefit, Usability, and Sensation. Comprehension is supposed to be a subjective measure of how well the participants understood what information the feedback was providing; Benefit asked about how much the feedback helped with completing the task; Usability asked whether the feedback was easy or difficult to use; and Sensation inquired about the pleasantness of the feedback. A similar extension of the TLX had already been used in a previous study [3].


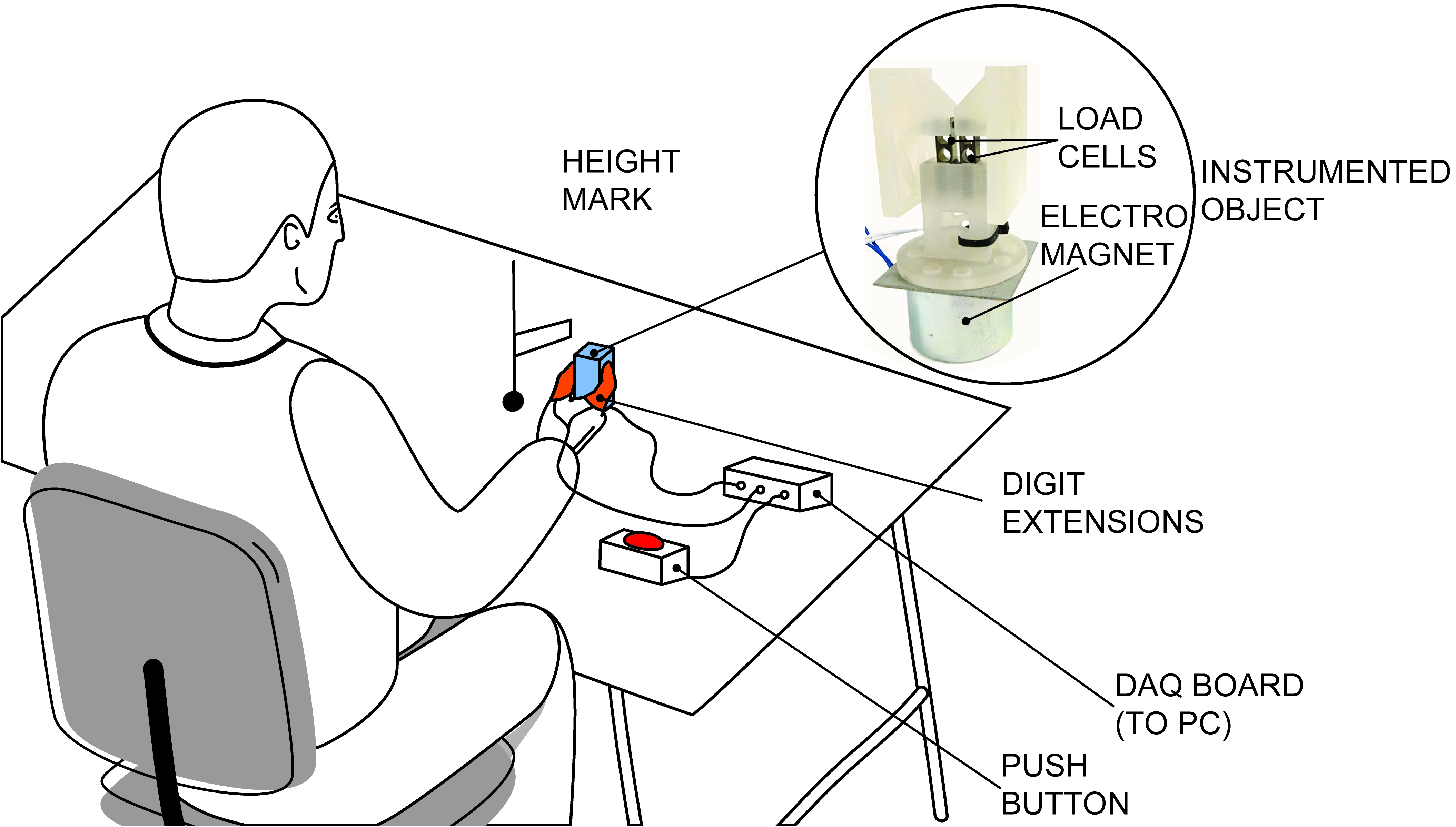


**S1 Fig. Schematic of the experimental setup.** This drawing shows the basic experimental setup.

## Supplementary Results


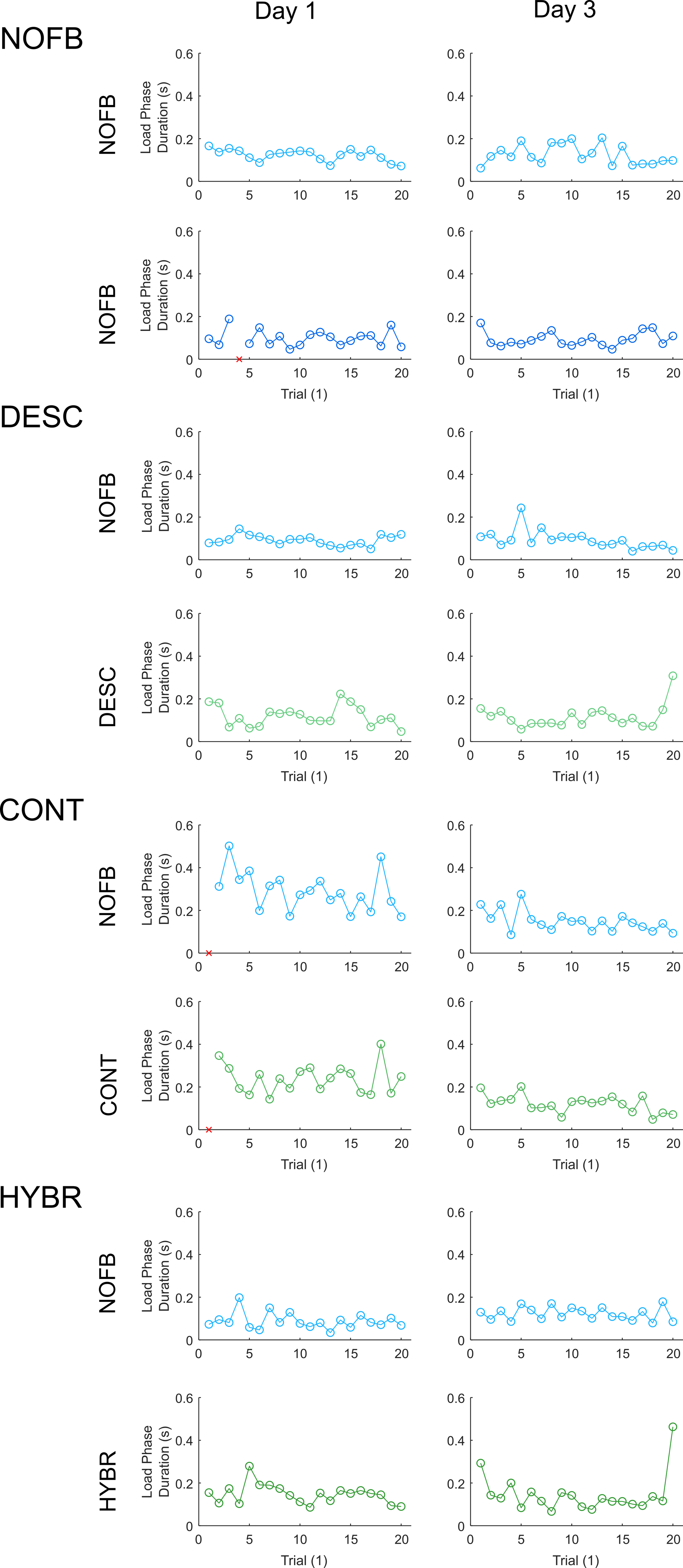


**S2 Fig.Example time series of the load phase duration by random subjects in Task 1 of Study 1.** The graphs show the load phase duration of each of the 20 trials in Task 1 for Participant 8 (NOFB), 5 (DESC), 6 (CONT), and 3 (HYBR) each without feedback and with feedback according to group, on day 1 (left column) and day 3 (right column). Missing trials are denoted as a red ‘x’.


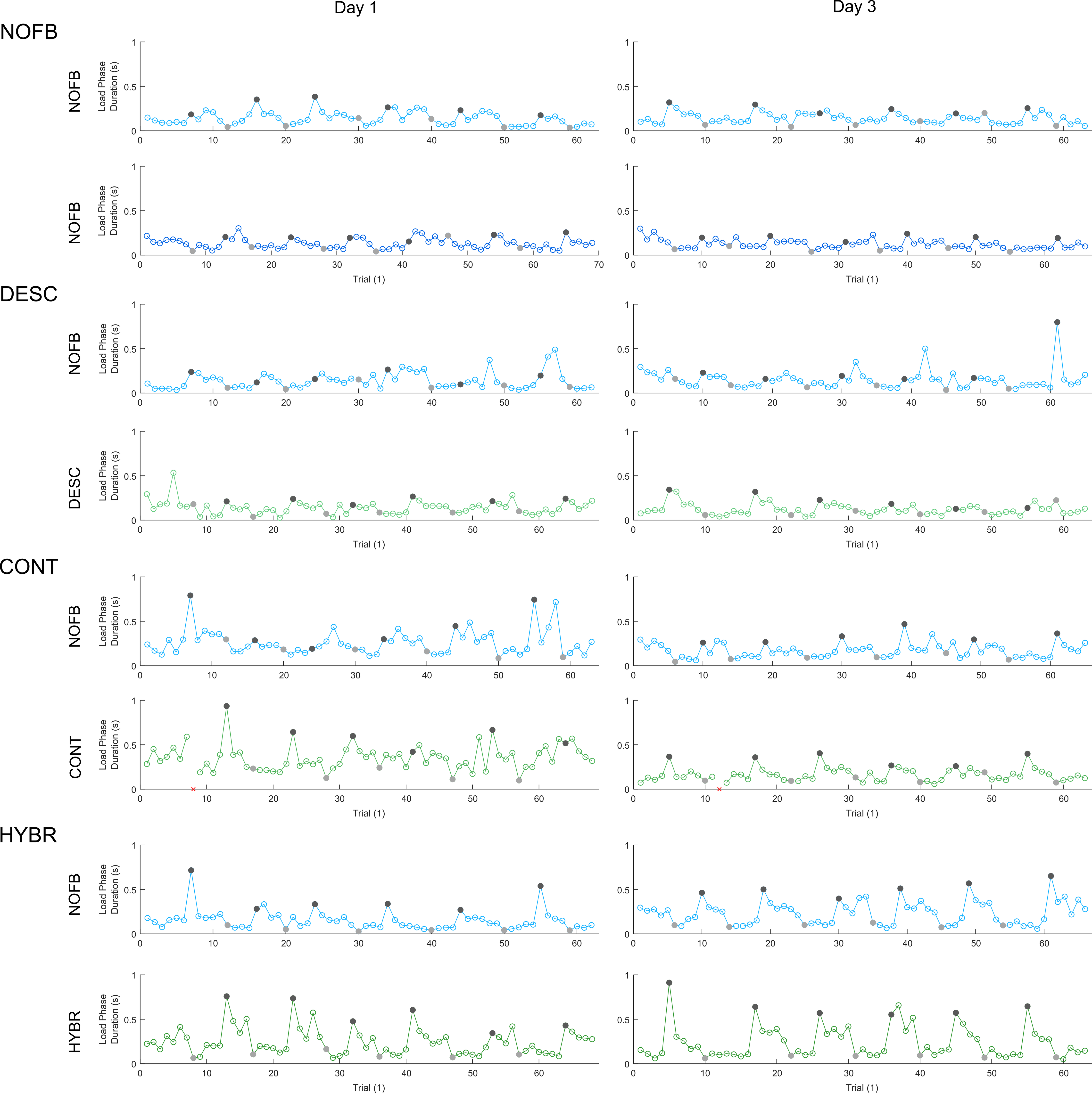


**S3 Fig. Example time series of the load phase duration by random subjects in Task 2 of Study 1.** The graphs show the load phase duration of each of the >60 trials in Task 2 for Participant 8 (NOFB), 5 (DESC), 6 (CONT), and 3 (HYBR) each without feedback and with feedback according to group, on day 1 (left column) and day 3 (right column). Trials where the weight suddenly changed from light to heavy are marked dark grey, changes from heavy to light are marked light grey.


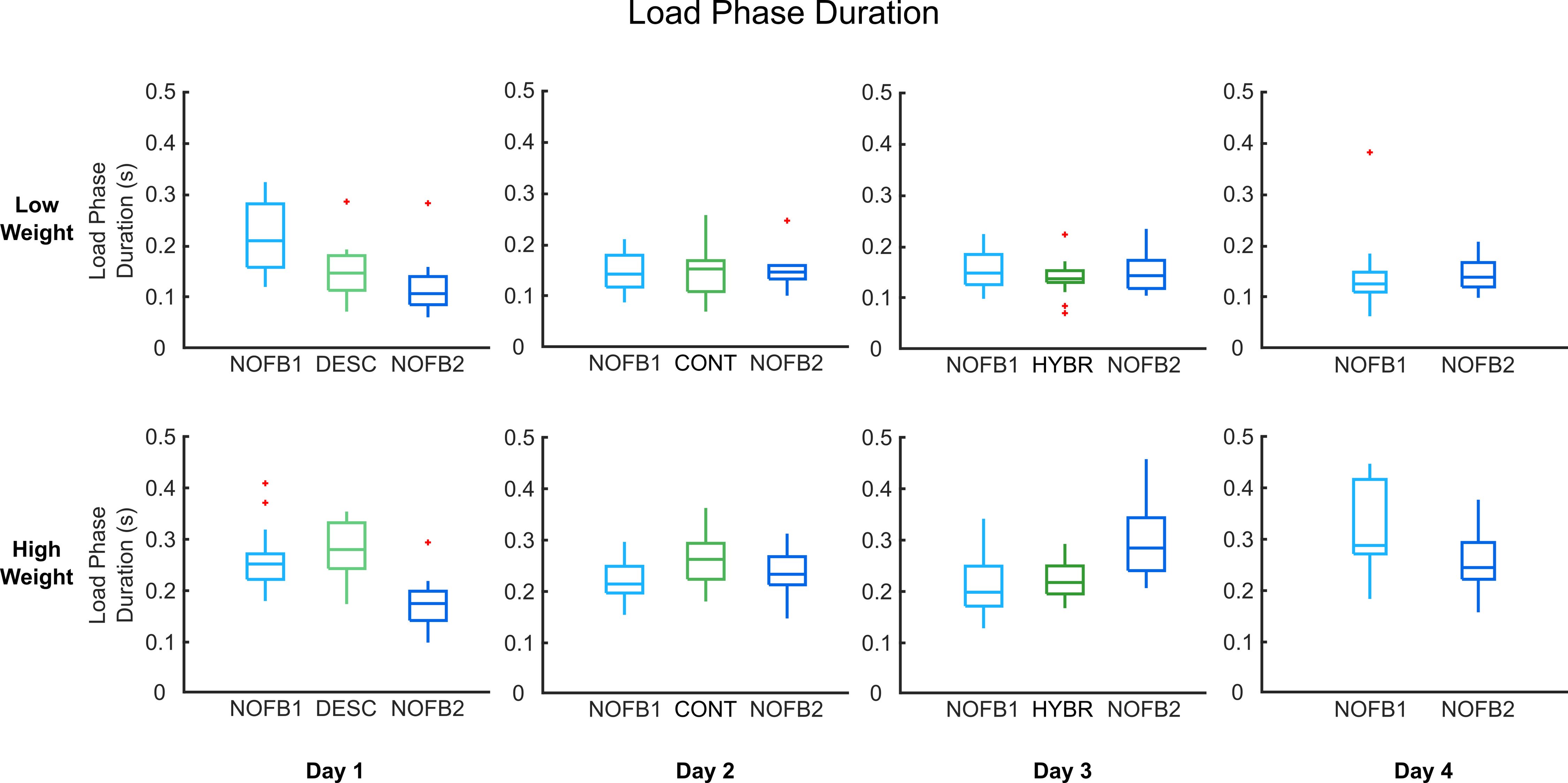


**S4 Fig.** **Boxplots of the load phase durations in Task 1 of Study 2.** Trials without feedback are displayed in blue, trials with feedback in green. It can easily be seen that most trials overlap to some extent. Boxplots display medians, 25^th^ and 75^th^ percentiles, the whiskers denote the most extreme datapoints that are not outliers. Red crosses denote outliers.


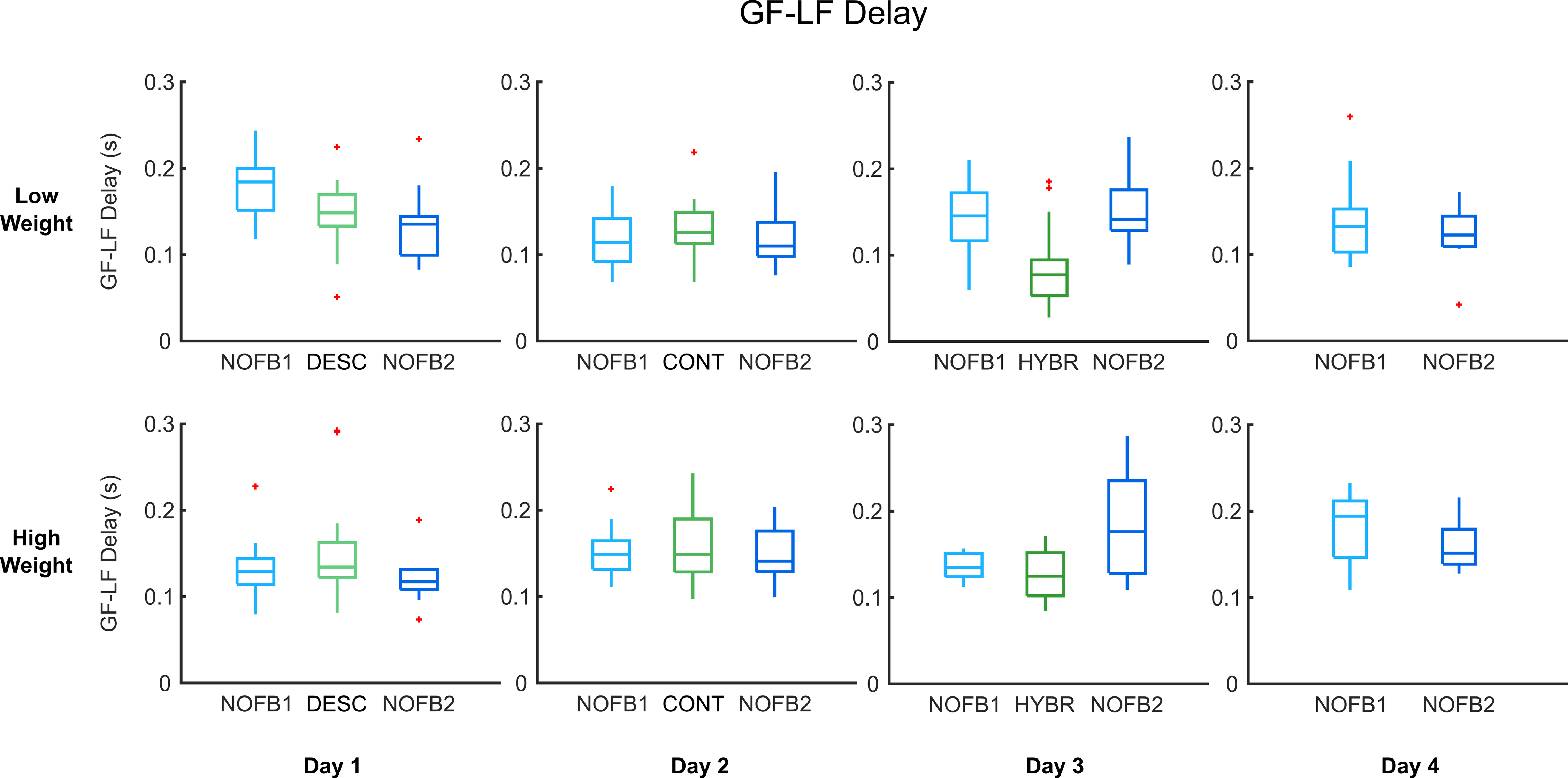


**S5 Fig.** **Boxplots of the GF-LF delays in Task 1 of Study 2.** Trials without feedback are displayed in blue, trials with feedback in green. It can easily be seen that most trials overlap to a large extent, with only one difference with HYBR and the lower weight. All graphs show boxplots (median and interquartile range); red crosses denote outliers.


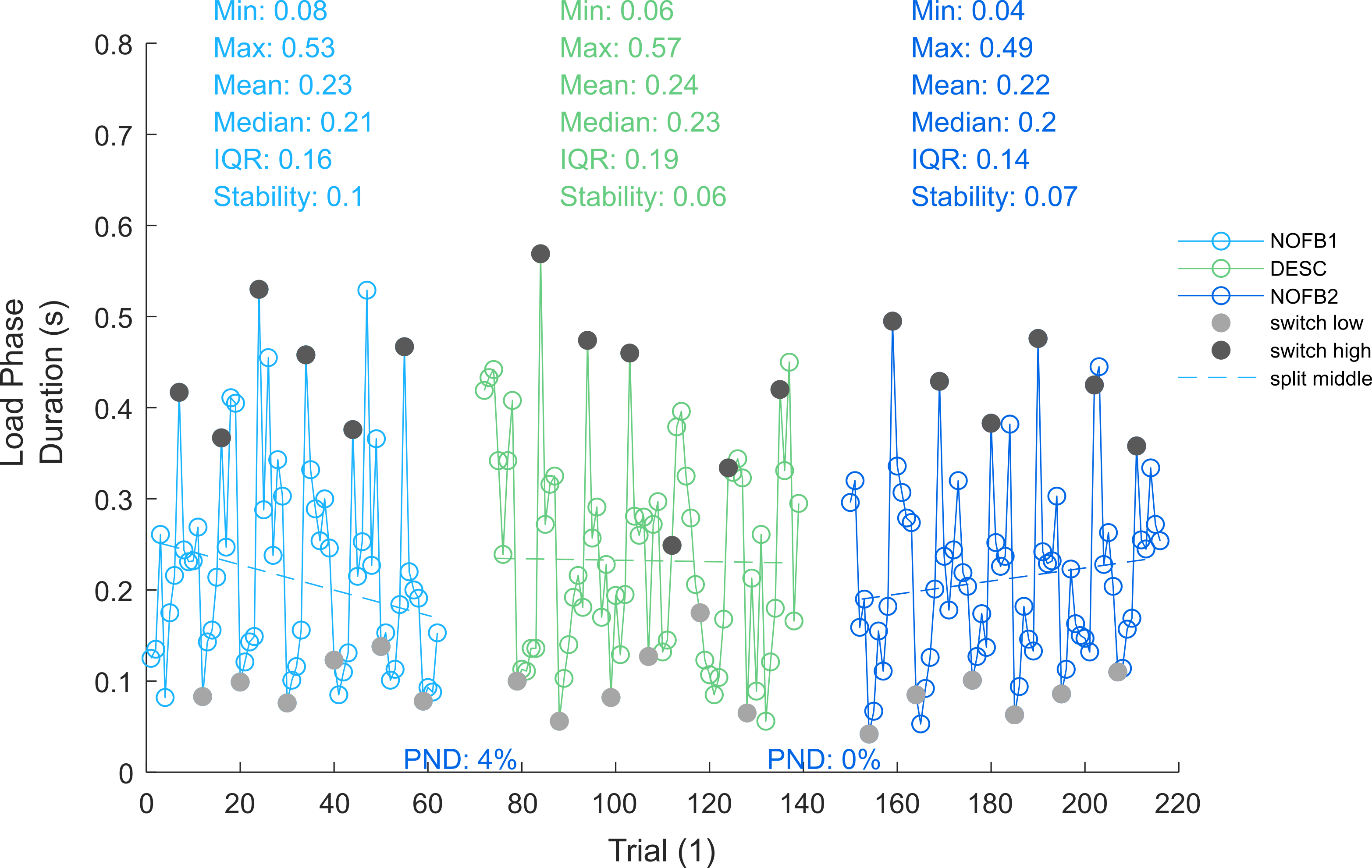


**S6 Fig. Example time series of the load phase duration with and without DESC feedback in Task 2 of Study 2.** The trials without feedback are presented in blue, the trials in green are with DESC feedback. This example shows that there is an almost complete overlap of trials without and with feedback. PND = Percentage of non-overlapping datapoints between two adjacent phases (in the desired direction, i.e., “DESC lower than NOFB”); IQR = interquartile range; Stability = percentage of datapoints that are within 15% of the median.

### Questionnaires


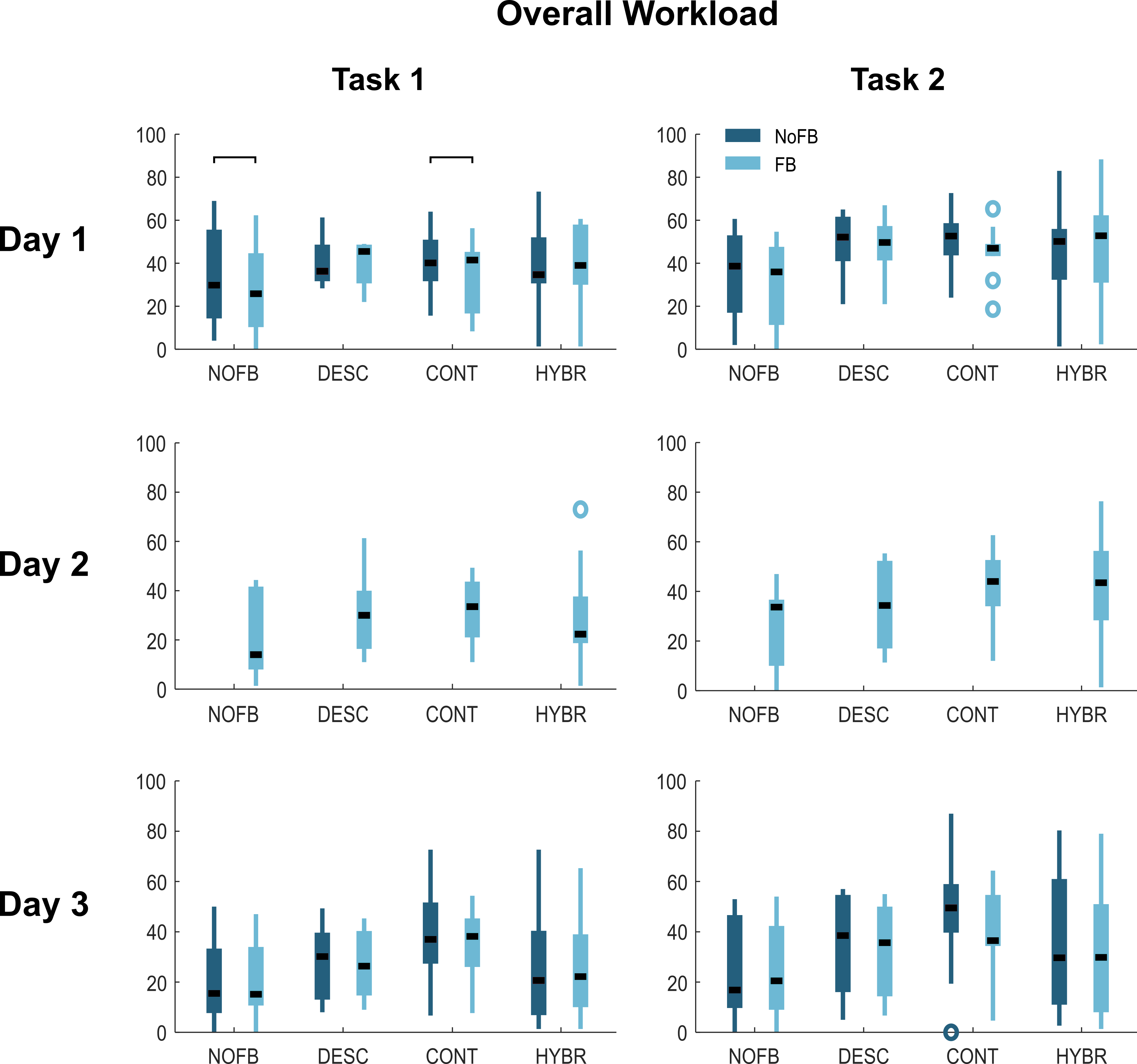


**S7 Fig. Overall Workload score of the NASA TLX for Tasks 1 and 2 of Study 1.** The overall workload is calculated from the weighted sub-scores of the TLX and attempts to describe the workload that participants experienced while performing Tasks 1 and 2, either without (dark blue) or with feedback (light blue). Horizontal bars denote significance (p < 0.05) between feedback conditions within each group on days 1 and 3.

#### Study 1

**Task 1**

There were some significant differences between groups when they did not receive feedback. This was expected as the NASA TLX offers a subjective evaluation of the task’s workload. Therefore, groups where not compared with each other when receiving feedback; rather, the scores within one group with and without feedback were compared.

Looking at raw scores, there is a small difference in Mental Demand for the CONT group without feedback and when receiving continuous feedback on day 1, suggesting that continuous feedback (median: 37.5, IQR: 30) may have lowered the Mental Demand slightly compared to no feedback (median: 60, IQR: 25). This is reflected in the Overall Workload (S7 Fig.). However, this difference dispersed by day 3 for mental demand and Overall Workload.

There is, further, a small difference in Physical Demand for the DESC group on day 3. Discrete feedback (median: 20, IQR: 25) may have slightly lowered the Physical Demand compared to no feedback (median: 37.5, IQR: 45). This difference is not reflected in the Overall Workload.

All other raw scores show no difference.

Regarding the extension of the questionnaire specifically designed to enquire about the feedback, we did not find any notable difference between the conditions with and without feedback (S8 Fig.).


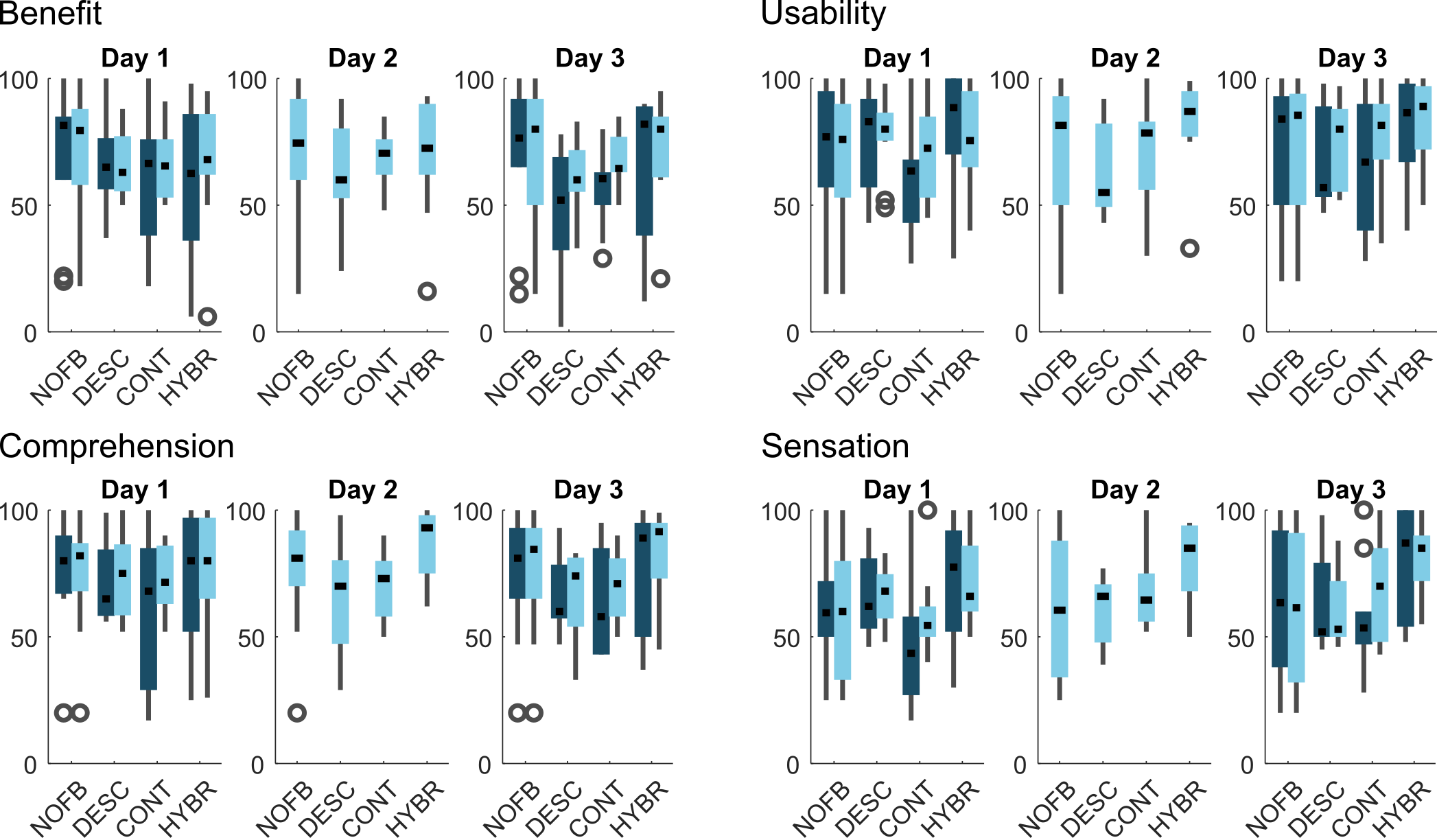


**S8 Fig. Results of the feedback extension of the questionnaire for Study 1, Task 1.** Participants were asked to rate the Benefit, Usability, their Comprehension, and the Sensation of the sensory feedback without (dark blue) and with (light blue) supplementary feedback. The scale is comparable to that of the TLX, ranging from 0 “worst” to 100 “best”. All graphs show boxplots (median and interquartile range), circles denote outliers.

**Task 2**

As can be seen in S7 Fig., there were no statistically significant differences between perceived Overall Workload with or without feedback in any of the feedback groups on any of the days.


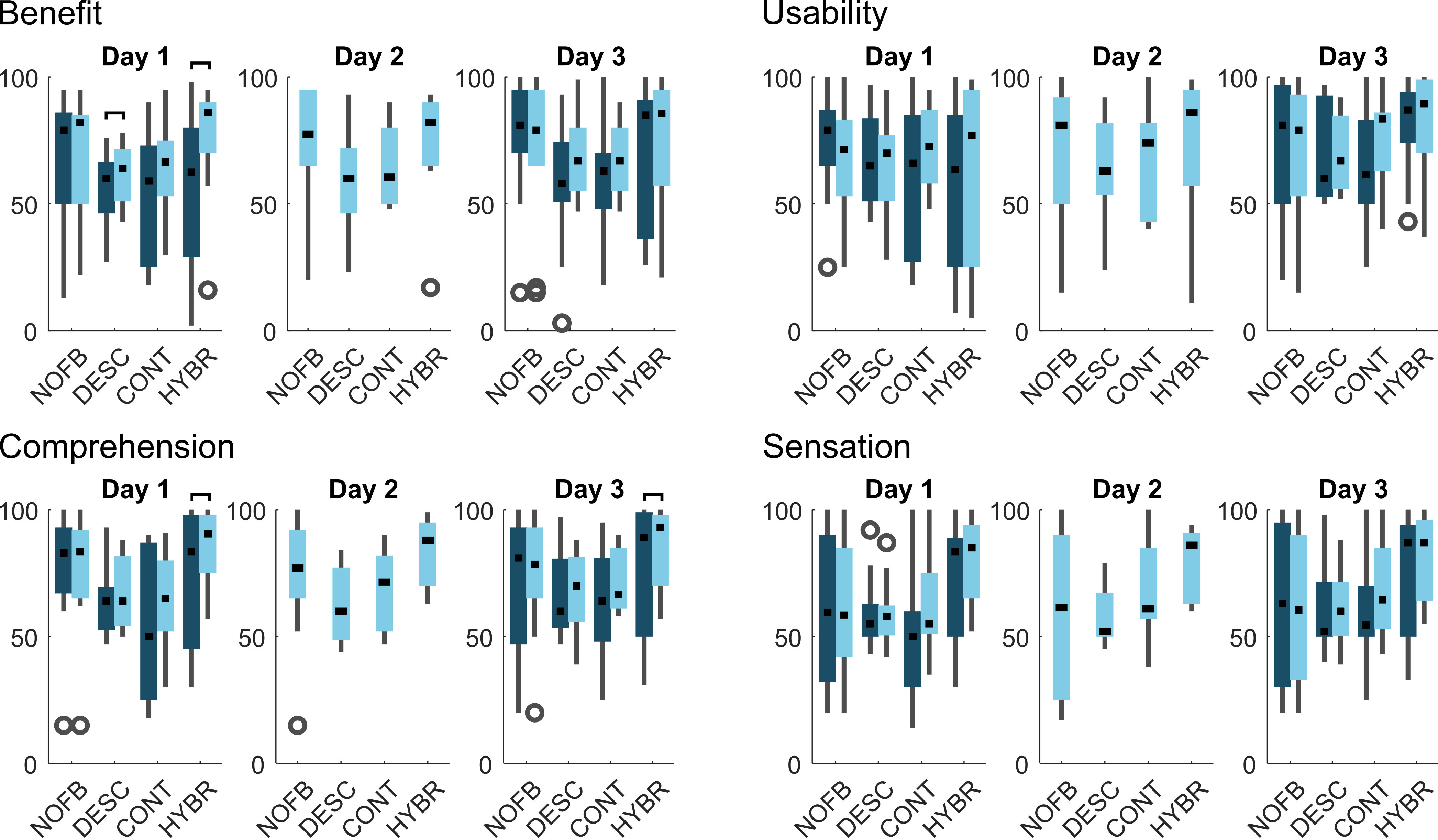


**S9 Fig. Results of the feedback extension of the questionnaire for Study 1, Task 2.** Participants were asked to rate the Benefit, Usability, their Comprehension, and the Sensation of the sensory feedback without (dark blue) and with (light blue) supplementary feedback. The scale is comparable to that of the TLX, ranging from 0 “worst” to 100 “best”. All graphs show boxplots (median and interquartile range), circles denote outliers. Significant differences (p<0.05) between feedback and no-feedback conditions within each day are denoted by horizontal bars.

The raw scores were normally distributed, and paired t-tests revealed significantly lower Effort and Frustration with CONT feedback than without on day 3 (Effort: NOFB median: 52.5, IQR: 30 vs. CONT median: 37.5, IQR: 25; p = 0.042; Frustration: NOFB median: 25, IQR: 50 vs. CONT median: 20, IQR: 30; p = 0.042).

With HYBR feedback, Physical Demand and Temporal Demand were rated significantly higher on day 1 than without feedback (Physical Demand: NOFB median: 52.5, IQR: 10 vs. HYBR median: 62.5, IQR: 20; p = 0.016; Temporal Demand: NOFB median: 22.5, IQR: 45 vs. HYBR median: 37.5, IQR: 50; p = 0.036).

For all other scores and for the DESC and NOFB groups, we found no significant differences.

In Task 2, DESC (p = 0.017) and HYBR (p = 0.006) feedback were perceived as providing significantly more benefit than no feedback, but only on the first day (S9 Fig.). Feedback from HYBR was also perceived as being better understood than the diffuse sensations that participants had without explicit feedback, both on days 1 (p = 0.031) and 3 (p = 0.031).

#### Study 2

**Task 1**

In study 2, again, we found no remarkable differences between any of the scores (S10 Fig.). In Task 1, the Overall Workload is nearly the same for all four days, with and without feedback.


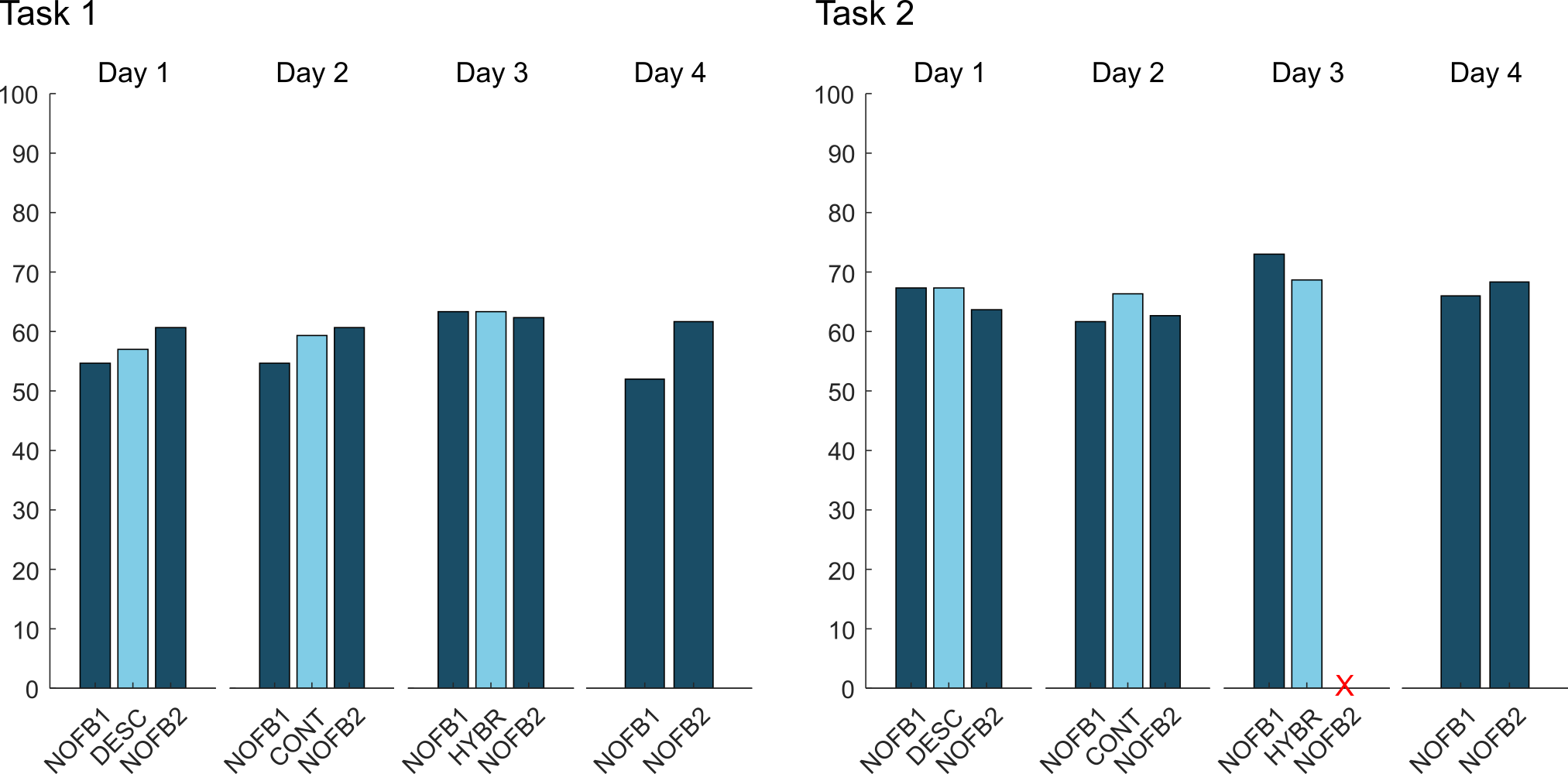


**S10 Fig. NASA TLX Overall Workload for Task 1 and 2 of Study 2.** The participant rated the Benefit, Usability, their Comprehension, and the Sensation of the sensory feedback without (dark blue) and with (light blue) supplementary feedback. The data for the second NOFB questionnaire on day 3 in Task 2 is missing.

Regarding the raw scores, there was a very slight decrease of Mental Demand with DESC compared to without FB (85 vs 90) and a very slight increase with CONT (85 vs 80). Physical Demand rose strongly over the three repetitions of the task on days 1, 2, and 4 (from 35/50/55 to 65/75/75, respectively). Frustration was rated low, but higher with CONT than without feedback (20 vs 10). There was no tangible difference in any of the other scores.

**Task 2**

Regrettably, the data for Task 2 for the second NOFB condition on day 3 was lost. The Overall Workload was comparable between feedback and no-feedback conditions.

The raw Mental Demand score with DESC was slightly higher than on the same day without feedback (NOFB1: 90, DESC: 95, NOFB2: 85). With CONT, the Physical Demand was slightly higher (NOFB1: 70, CONT: 75, NOFB2: 65), and so was the Frustration score (10, 15, 10). The latter seemed to decrease with HYBR (25 vs. 15), but since data for NOFB2 is missing, we cannot confirm whether this was not simply a generally decreasing trend. Interestingly, Effort was rated very highly on the first repetition of the first day but then decreased and became rather stable for the rest of the experiment (from 95 to 75-80).

It may appear in Task 1 and 2 that the subjective experience with HYBR feedback was slightly worse. However, also the experience without feedback on that day was worse than on other days. And since GN reported that her constitution varies strongly from day to day, she may just have felt worse generally on day 3, irrespective of our experiment.


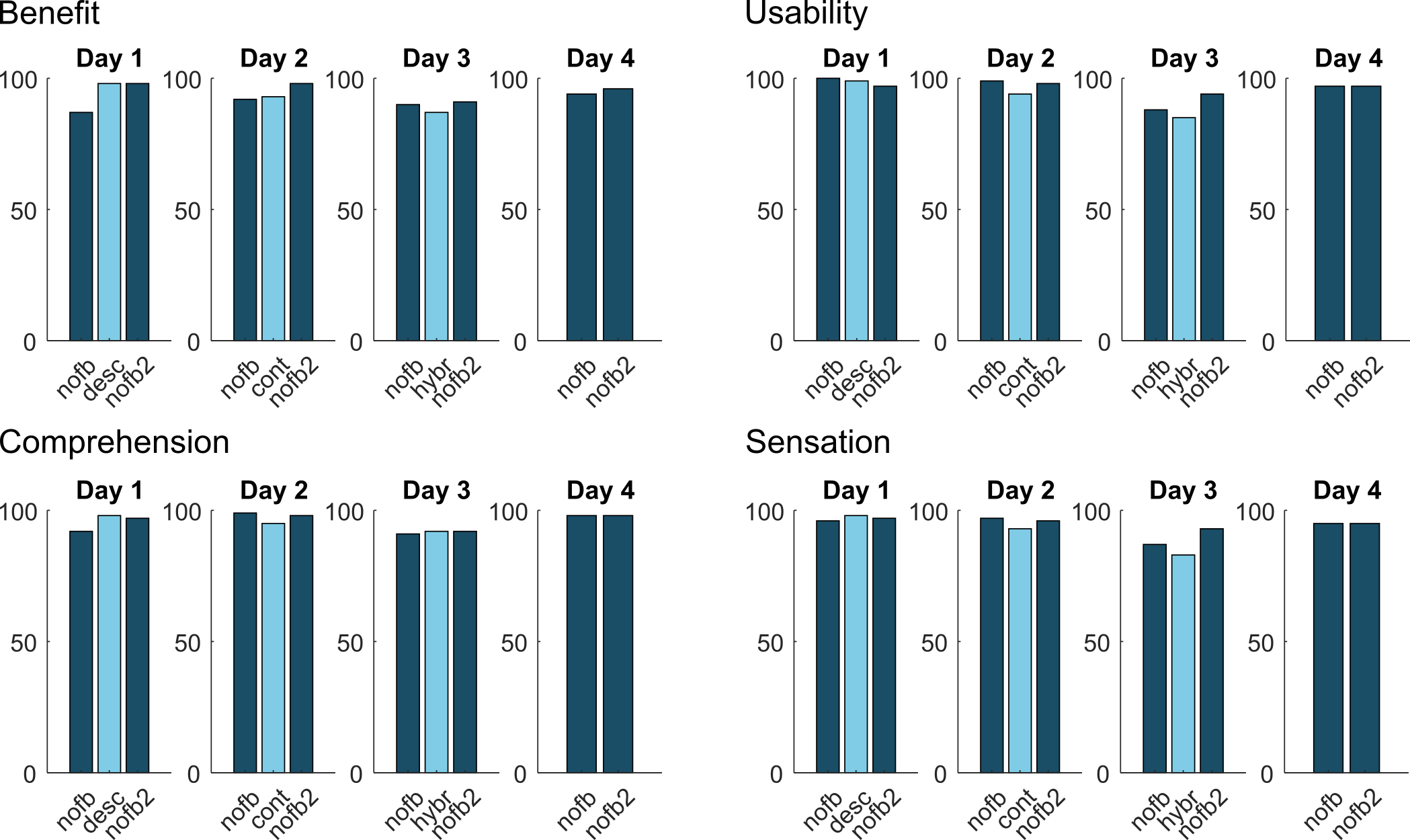


**S11 Fig.** **Results of the feedback extension of the questionnaire for Study 2, Task 1.** The participant rated the Benefit, Usability, their Comprehension, and the Sensation of the sensory feedback without (dark blue) and with (light blue) supplementary feedback.


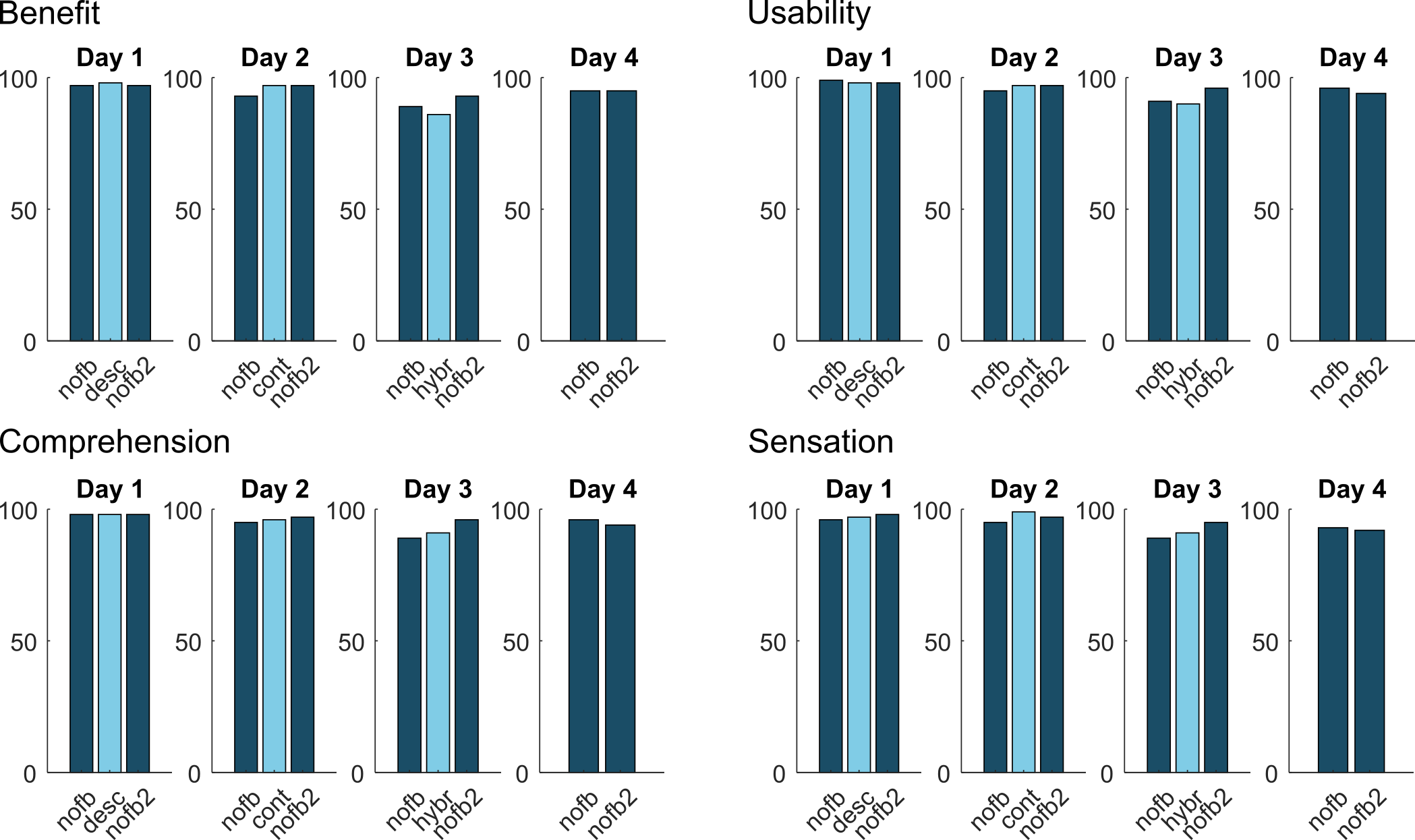


**S12 Fig. Results of the feedback extension of the questionnaire for Study 2, Task 2.** The participant rated the Benefit, Usability, their Comprehension, and the Sensation of the sensory feedback without (dark blue) and with (light blue) supplementary feedback.

## Supplementary references

1. Hart SG, Staveland LE. Development of NASA-TLX (Task Load Index): Results of Empirical and Theoretical Research. Adv Psychol. 1988;52: 139–183. doi:10.1016/S0166-4115(08)62386-9

2. Hart SG. NASA-TASK LOAD INDEX (NASA-TLX); 20 YEARS LATER. Proc Hum Factors Ergon Soc 50th Annu Meet. 2006; 904–908. doi:10.1037/e577632012-009

3. Markovic M, Schweisfurth MA, Engels LF, Bentz T, Wüstefeld D, Farina D, et al. The clinical relevance of advanced artificial feedback in the control of a multi-functional myoelectric prosthesis. J Neuroeng Rehabil. 2018;15: in press. doi:10.1186/s12984-018-0371-1
